# Isolation and Characterization of vB_ArS-ArV2 – First *Arthrobacter* sp. Infecting Bacteriophage with Completely Sequenced Genome

**DOI:** 10.1371/journal.pone.0111230

**Published:** 2014-10-21

**Authors:** Eugenijus Šimoliūnas, Laura Kaliniene, Miroslav Stasilo, Lidija Truncaitė, Aurelija Zajančkauskaitė, Juozas Staniulis, Juozas Nainys, Algirdas Kaupinis, Mindaugas Valius, Rolandas Meškys

**Affiliations:** 1 Department of Molecular Microbiology and Biotechnology, Institute of Biochemistry, Vilnius University, Vilnius, Lithuania; 2 Laboratory of Plant Viruses, Institute of Botany, Nature Research Centre, Vilnius, Lithuania; 3 Department of Eukaryote Gene Engineering, Institute of Biotechnology, Vilnius University, Vilnius, Lithuania; 4 Proteomics Centre, Institute of Biochemistry, Vilnius University, Vilnius, Lithuania; Loyola University Medical Center, United States of America

## Abstract

This is the first report on a complete genome sequence and biological characterization of the phage that infects *Arthrobacter*. A novel virus vB_ArS-ArV2 (ArV2) was isolated from soil using *Arthrobacter* sp. 68b strain for phage propagation. Based on transmission electron microscopy, ArV2 belongs to the family *Siphoviridae* and has an isometric head (∼63 nm in diameter) with a non-contractile flexible tail (∼194×10 nm) and six short tail fibers. ArV2 possesses a linear, double-stranded DNA genome (37,372 bp) with a G+C content of 62.73%. The genome contains 68 ORFs yet encodes no tRNA genes. A total of 28 ArV2 ORFs have no known functions and lack any reliable database matches. Proteomic analysis led to the experimental identification of 14 virion proteins, including 9 that were predicted by bioinformatics approaches. Comparative phylogenetic analysis, based on the amino acid sequence alignment of conserved proteins, set ArV2 apart from other siphoviruses. The data presented here will help to advance our understanding of *Arthrobacter* phage population and will extend our knowledge about the interaction between this particular host and its phages.

## Introduction

*Arthrobacter* sp. strains are widely distributed in the environment and have been found to be among the predominant members of culturable aerobic soil bacteria [1]. The genus *Arthrobacter* includes a group of catalase-positive, strictly aerobic, sporogenous rod-shaped coryneform bacteria with a high mol% GC DNA composition (generally ranging from 59 to 66%) and A-type (A3a or A4a) peptidoglycans with L-lysine as the dibasic amino acid [2]. The environmental prevalence of *Arthrobacter* sp. strains is considered to be due to their nutritional versatility and their pronounced resistance to desiccation, long-term starvation and environmental stress [1]. It is unsurprising that strains of the genus *Arthrobacter* are phenotypically heterogeneous and have been isolated from distinct sources, such as soil [3], wastewater sediments [4], clinical specimens [5], animals [6], phyllosphere [7], paintings [8], cheese [9] and air [10]. Moreover, *Arthrobacter* spp. have been found in extreme environments, such as the Arctic/Antarctic waters and sediments [11], chemically contaminated sites [12] and radioactive environments [13]. It was reported that certain species of the genus *Arthrobacter* have the capacity to degrade various difficult-to-degrade chemical substrates [14] and, in a few cases, exhibit denitrification activity [15]. To summarize, bacteria of the genus *Arthrobacter* are thought to play a significant role in many ecosystems and affect human welfare.

Although different *Arthrobacter* strains have been the subject of extensive studies, relatively little is known about their predators in nature – bacteriophages, and only a limited number of reports on *Arthrobacter* phages have been published thus far. Robinson and Corke [16] observed plaques when soil perfusates were plated with *Arthrobacter*. In addition, some authors [17], [18] investigated the use of bacterial viruses in the phage typing of soil *Arthrobacter*. Most of publications, however, described only the isolation and/or partial characterization of bacteriophages active on laboratory strains and soil isolates of *Arthrobacter* [18], [19], [20], [21], [22], [23], [24], and only a few studies provided more detailed characterizations of *Arthrobacter* phages [25], [26], [27]. The majority of *Arthrobacter* phages described to date belong to the family *Siphoviridae* [28]. To our knowledge, only phages AN25S-1 and AN29R-2 [18] showed morphological characteristics of podoviruses. Interestingly, while most reports about *Arthrobacter* phages were published during the seventies or eighties of the last century, none of the published *Arthrobacter* phages was tested regarding genome sequence/organisation or similarity to already known viruses (see Table S1).

This study represents the first detailed characterization of an *Arthrobacter* bacteriophage with a complete DNA sequence and annotation. Phage vB_ArS-ArV2 (ArV2) was isolated from soil and characterized with respect to morphology and biological properties. The data of genomic, proteomic and phylogenetic analyses indicate that ArV2 has no close relatives within the family *Siphoviridae* of tailed bacteriophages. Given that arthrobacterial viruses have never been investigated at the genomic level to date, the results provided in this report finally offer a glimpse into the biology of bacteriophages that infect *Arthrobacter*.

## Materials and Methods

### Phages and bacterial strains

This study does not require an ethics statement (N/A). Phage ArV2 was isolated from soil samples collected on private land in Vilnius, Lithuania (54.839573, 25.369245). The person, who should be contacted for future permissions is Dr. Rolandas Meskys (rolandas.meskys@bchi.vu.lt). *Arthrobacter* sp. strain 68b was used as the host for phage propagation and phage growth experiments. Bacterial strains used in this study for host range determination are described in Table 1. For phage experiments, bacteria were cultivated in Luria-Bertani broth (LB) or LB agar at 30°C. Bacterial growth was monitored turbidimetrically by reading OD_600_. An OD_600_ of 1.0 corresponded to 9×10^8^ *Arthrobacter* sp. 68b cells/ml.

**Table 1:** Bacterial strains used in this study.

Strain	Relevant characteristics	Source or reference
*Acinetobacter baumannii* #46		Prof. E. Suziedeliene
*Acinetobacter* gen. sp. 13#23		Prof. E. Suziedeliene
*Arthrobacter chlorophenolicus* DSM 12829	type strain	DSMZ
*Arthrobacter crystallopoetes* DSM 20117	type strain	DSMZ
*Arthrobacter defluvii* DSM 18782	type strain	DSMZ
*Arthrobacter globiformis* DSM 20124	type strain	DSMZ
*Arthrobacter histidinolovorans* DSM 20115	type strain	DSMZ
*Arthrobacter ilicis* DSM 20138	type strain	DSMZ
*Arthrobacter methylotrophus* DSM 14008	type strain	DSMZ
*Arthrobacter nicotinovorans* DSM 420	type strain	DSMZ
*Arthrobacter nitroguajacolicus* DSM 15232	type strain	DSMZ
*Arthrobacter oxydans* DSM 20119	type strain	DSMZ
*Arthrobacter* sp. 68b	environmental isolate	[29]
*Arthrobacter* sp. 68m	environmental isolate	laboratory collection
*Arthrobacter* sp. 83b	environmental isolate	laboratory collection
*Arthrobacter* sp. 85	environmental isolate	laboratory collection
*Arthrobacter* sp. 94	environmental isolate	laboratory collection
*Arthrobacter* sp. 96	environmental isolate	laboratory collection
*Arthrobacter* sp. 25DMP1	environmental isolate	[14]
*Arthrobacter* sp. 25DOT1	environmental isolate	[14]
*Arthrobacter* sp. PY11	environmental isolate	[30]
*Arthrobacter* sp. PY21	environmental isolate	[31]
*Arthrobacter* sp. PY22	environmental isolate	[31]
*Arthrobacter* sp. VP3	environmental isolate	[30]
*Arthrobacter* sp. PRH1	environmental isolate	[31]
*Arthrobacter* sp. VM22	environmental isolate	[30]
*Arthrobacter* sp. IN13	environmental isolate	[32]
*Arthrobacter ureafaciens* DSM 20126	type strain	DSMZ
*Citrobacter freundii*		Prof. E. Suziedeliene
*Enterobacter cloacae*		Prof. E. Suziedeliene
*Erwinia carotovora* 8982		Prof. E. Suziedeliene
*Escherichia coli* B^E^	sup^0^	Dr. L. W. Black
*Escherichia coli* BL21 (DE3)	F^−^ dcm ompT hsdS(rB^−^mB^−^) gal λ(DE3)	Avidis
*Escherichia coli* DH5α	F^−^ endA1 glnV44 thi-1 recA1 relA1 gyrA96 deoR nupG Φ80dlacZΔM15 Δ(lacZYA-argF)U169, hsdR17(r_K_^−^ m_K_^+^), λ–	Pharmacia
*Escherichia coli* DH10β	F^−^ endA1 recA1 galE15 galK16 nupG rpsL ΔlacX74 Φ80lacZΔM15 araD139 Δ(ara,leu)7697 mcrA Δ(mrr-hsdRMS-mcrBC) λ^−^	Invitrogen
*Escherichia coli* DH5α	F^−^ endA1 glnV44 thi-1 recA1 relA1 gyrA96 deoR nupG Φ80dlacZΔM15 Δ(lacZYA-argF)U169, hsdR17(r_K_^−^ m_K_^+^), λ–	Pharmacia
*Klebsiella pneumoniae* 279		Prof. E. Suziedeliene
*Klebsiella* sp. KV-3	Phage host, veterinary isolate, Amp^r^, Str^r^, Tet^r^, Kan^s^, Gm^s^, Nc^s^,Cl^r/s^	[33]
*Pseudomonas aeruginosa* PAO1		Prof. E. Suziedeliene
*Salmonella enterica* ser. Typhimurium 292		Prof. E. Suziedeliene

### Phage techniques

Bacteriophages active on *Arthrobacter* spp. were searched for in various samples of soil (collected from the different sites in Lithuania). Soil samples (1–5 g) were shaken for 1 h in 10 ml of LB broth followed by low-speed centrifugation at 5000 rpm for 15 min. The supernatant fluid was sequentially filtered through sterile 0.45 and 0.2 µm membrane filters and assayed for plaque-forming units by the soft agar overlay method described by Adams [34] with minor modifications. Briefly, 0.1 ml of diluted phage suspension or clarified environmental sample was mixed with 0.5 ml of indicator cells (OD_600_ – 1). The mixture then was added to 2.5 ml of 0.5% (w/v) soft agar and poured over the 1.2% LB agar plate as a uniform layer. The plates were incubated 24–48 h at 30°C before the enumeration of plaques. Bacteriophage culture was purified by performing five consecutive transfers of phage from individual plaques to new bacterial cell lawns. Phage ArV2 propagation was performed using a standart procedure with few modifications using *Arthrobacter* strain 68b as a host. Briefly, phage particles were subsequently collected by adding 5 ml of LB broth to the surface of each plate. The top agar was scraped off and the suspension recovered. After 30 min. of incubation at 4°C with mild stirring the mixture was centrifuged at 6000 rpm for 15 min. The phage-containing supernatant was decanted and the phage was concentrated by high-speed centrifugation at 30000 rpm for 3 h. The resulting pellets were suspended in PB buffer (70 mM NaCl, 10 mM MgSO_4_, 50 mM Na_2_HPO_4_, 30 mM KH_2_PO_4_). To avoid bacterial DNA contamination, DNase I was added to the phage suspension, and the sample was incubated 1 h at 37°C. Further purification was performed using a CsCl step gradient [35] as described previously [33]. The adsorption tests were carried out as described by Kropinski et al. [36]. Meanwhile determination of the efficiency of plating (e.o.p.) was performed as described by Kaliniene et al. [37]. High-titer phage stocks were diluted and plated in duplicate. Plates incubated at 18, 21, 22, 24, 26, 27, 28, 30, 32, 34 and 37°C were read after 18–96 hours of incubation. The temperature at which the largest number of plaques was formed was taken as the standard for the e.o.p. calculation.

### Transmission electron microscopy (TEM)

CsCl density gradient-purified phage particles were diluted to approximately 10^11^ PFU/ml with distilled water, 5 µl of the sample was directly applied on the carbon-coated nitrocellulose grid, excess liquid was drained with filter paper before staining with two successive drops of 2% uranyl acetate (pH 4.5), dried and examined in Morgagni 268(D) transmission electron microscope (FEI, Oregon, USA).

### DNA isolation and restriction analysis

Aliquots of phage suspension (10^11^–10^12^ PFU/ml) were subjected to phenol/chloroform extraction and ethanol precipitation as described by Carlson and Miller [38]. Isolated phage DNA was subsequently used in restriction analysis, for PCR or was subjected to genome sequencing. Restriction digestion was performed with BamHI, EcoRI, EcoRII, EcoRV, HindIII, KpnI, MboI, NheI, NotI, PstI, PvuI, SnaBI, SspI, VspI and XbaI restriction endonucleases (Fermentas) according to the supplier's recommendations. DNA fragments were separated by electrophoresis in a 0.8% agarose gel containing ethidium bromide. Restriction analysis was performed in triplicate to confirm the results.

### Genome sequencing and analysis

The complete genome sequence of ArV2 was determined using Illumina DNA sequencing technology at BaseClear, the Netherlands. Open reading frames (ORFs) were predicted with Glimmer v2.02 (http://nbc11.biologie.uni-kl.de) and Geneious v5.5.6. (Société Geneious, Colombes, France). Analysis of the genome sequence was performed using the Fasta-Protein, Fasta-Nucleotide, Fasta- Genome, BLAST2, PSI-Search, Transeq, ClustalW2, (http://www.ebi.ac.uk), HHsearch1.6 (http://toolkit.tuebingen.mpg. de/hhpred), COMA (http://bioinformatics.ibt.lt:8085/coma), Sequence editor (http://www.fr33.net/seqedit.php) programs and tRNAscan-SE 1.21 (http://lowelab.ucsc.edu/tRNAscan-SE/) was used to search for tRNAs. Phylogenetic and molecular evolutionary analyses were conducted using MEGA version 5 [39]. The physical location and nature of the genome ends were determined by combining the restriction analysis and subsequent sequencing directly from the genome ends. The sequencing reactions were carried out using a CycleReader DNA sequencing kit (Fermentas, Lithuania). The oligonucleotide primers (ArV2_F 5′- GACTAGCGCACCGACTATGCAAC and ArV2_R 5′- GCATCACTCAAGGACATC) for the sequencing reactions were 5′– end labeled using T4 polynucleotide kinase (Thermo Fisher Scientific, Lithuania) with [γ^32^P]ATP (PerkinElmer, USA).

### Analysis of Structural Proteins

#### SDS-PAGE

CsCl-purified phage particles (10^11^ pfu/ml) were resuspended in a buffer containing 60 mM Tris–HCl (pH 6.8), 1% SDS (w/v), 1% 2-mercaptoethanol (v/v), 10% glycerol (v/v) and 0.01% bromophenol blue (w/v). The samples were boiled for 3 min and separated on 12% SDS PAGE following the method described by Laemmli [40]. Protein bands were visualized by staining the gel with PageBlue Protein Staining Solution (Thermo Fisher Scientific, Vilnius, Lithuania).

#### Filter-aided protein sample preparation (FASP) for mass spectrometry analysis

CsCl-purified phage particles were concentrated on Amicon Ultra-0.5 mL 30 kDa centrifugal filter unit and were denatured in 8 M urea, 100 mM DTT solution with continuous rotation at 800 rpm in the temperature controlled shaker for 3 hours at 37°C.

Trypsin digestion was performed according to a modified FASP protocol as described by Wisniewski et al. [41]. Briefly, phage particles were washed with buffer containing 8 M urea. The proteins were alkylated using iodoacetamide. Buffer was exchanged by washing twice with 50 mM NH_4_HCO_3_, and proteins digested overnight with TPCK Trypsin 20233 (Thermo Scientific, USA). Then peptides were recovered by centrifugation and washed twice with 50% CH_3_CN. Afterwards, samples were combined, acidified, lyophilized, redissolved in 0.1% formic acid and analysed by mass spectrometry.

#### In-gel protein digestion for mass spectrometry analysis

In-gel trypsin digestion was done according to a protocol described by Hellman et al. [42] with minor modifications. Briefly, gel slices were destained with 200 µl of 50 mM ammonium bicarbonate in 50% CH_3_CN, vacuum-dried, rehydrated in 50 µl (20 µg ml^−1^) of trypsin TPCK Trypsin 20233 (Thermo Scientific, USA) containing 25 mM NH_4_HCO_3_ and incubated overnight at 37°C. The peptides were extracted from the gel using 100 µl of CH_3_CN for 30 min. Next, gel pieces were washed with (100 µl) of 1% formic acid for 10 min. Extraction procedure was finished by adding 100 µl of CH_3_CN. The peptides from all extractions were combined, acidified, concentrated by vacuum drying, resuspended in 40 µl 0.1% formic acid and then used for mass spectrometry analysis.

#### Liquid chromatography and mass spectrometry

Liquid chromatography (LC) separation of trypsin cleaved peptides was performed with nanoAcquity UPLC system (Waters Corporation, UK). Peptides were loaded on a reversed-phase trap column PST C18, 100Å, 5 µm, 180 µm×20 mm (Waters Corporation, UK) at a flow-rate of 15 µl/min using loading buffer of 0.1% formic acid and subsequently separated on HSS-T3 C18 1.8 µm, 75 µm×250 mm analytical column (Waters Corporation, UK) in 30 min linear gradient (A: 0.1% formic acid, B: 100% CH3CN and 0.1% formic acid) for in-gel protein trypsin digested samples or 60 min for FASP material at a flow rate of 300 nl per min. The analytical column temperature was kept at 40°C.

The nano-LC was coupled online through a nanoESI 7 cm length, 10 mm tip emitter (New Objective, USA) with HDMS Synapt G2 mass spectrometer (Waters Corporation, UK). Data were acquired using Masslynx version 4.1 software (Waters Corporation, UK) in positive ion mode. LC-MS data were collected using data independent acquisition (DIA) mode MS^E^ (for in-gel digested proteins) or MS^E^ in combination with online ion mobility separations (for FASP material).

The trap collision energy of mass spectrometer was ramped from 18 to 40 eV for high-energy scans in MS^E^ mode. The trap and transfer collision energy for high-energy scans in HDMS mode was ramped from 4 to 5 eV and from 27 to 50 eV. For both analyses, the mass range was set to 50–2,000 Da with a scan time set to 0.9 second. A reference compound [Glu1]-Fibrinopeptide B (Waters Corporation, UK) was infused continuously (500 fmol/µl at flow rate 500 nl per min) and scanned every 30 seconds for on-line mass spectrometer calibration purpose.

#### Data Processing, Searching and Analysis

Raw data files were processed and searched using ProteinLynx Global SERVER (PLGS) version 2.5.2 (Waters Corporation, UK). The following parameters were used to generate peak lists: (i) minimum intensity for precursors was set to 100 counts, (ii) minimum intensity for fragment ions was set to 30 counts, (iii) intensity was set to 500 counts. Processed data was analysed using trypsin as the cleavage protease, one missed cleavage was allowed and fixed modification was set to carbamidomethylation of cysteines, variable modification was set to oxidation of methionine. Minimum identification criteria included 2 fragment ions per peptide, 5 fragment ions per protein and minimum of 2 peptides per protein. The false discovery rate (FDR) for peptide and protein identification was determined based on the search of a reversed database, which was generated automatically using PLGS when global false discovery rate was set to 4%.

### Nucleotide sequence accession numbers

The complete genome sequence of *Arthrobacter* bacteriophage ArV2 was deposited in the EMBL nucleotide sequence database under accession number KF692088.

## Results and Discussion

### Virion morphology

**Figure 1:**
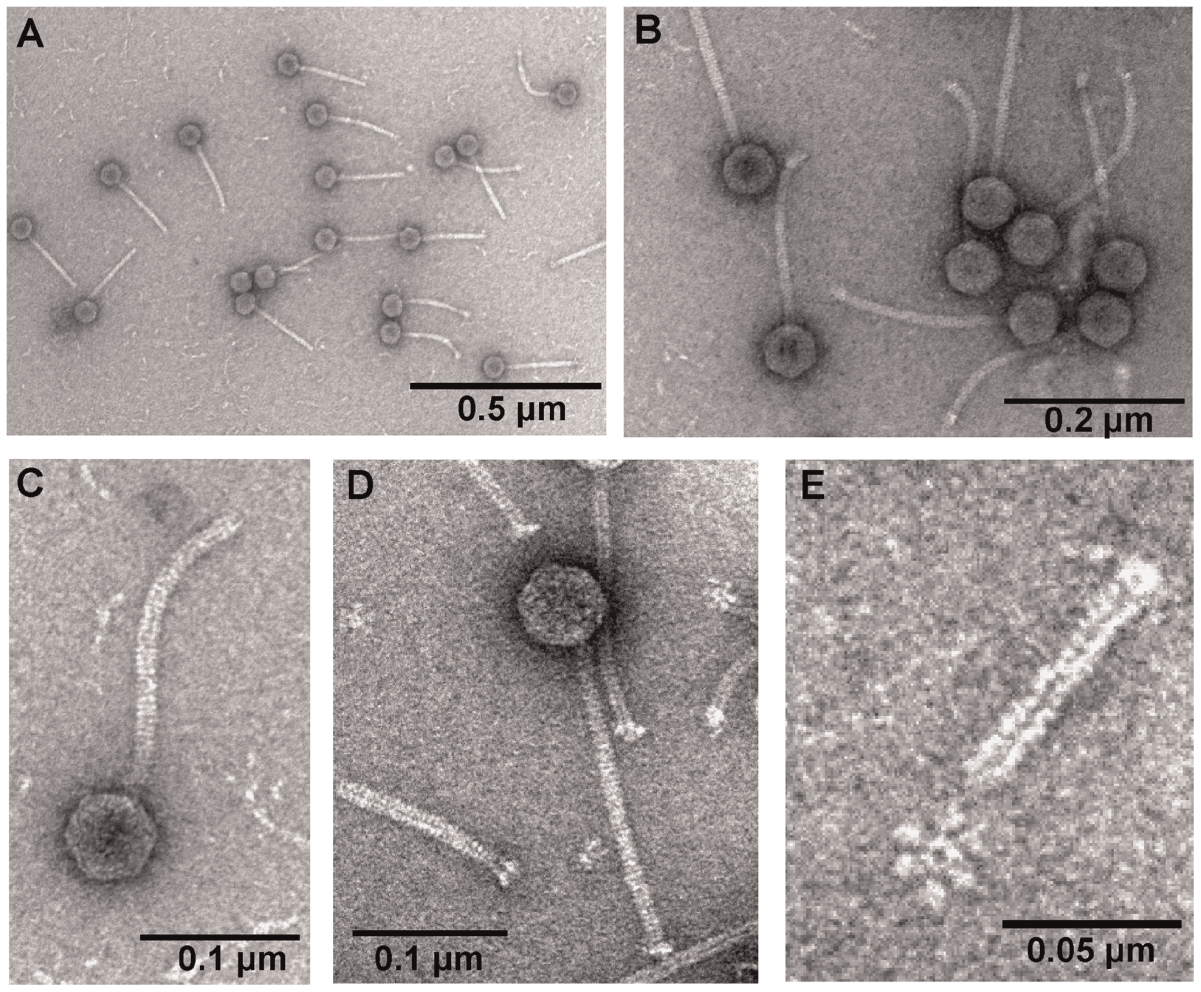
Electron micrographs of *Arthrobacter* phage ArV2.

TEM observations of ArV2 (Fig. 1) revealed a particle that fits B1 morphotype in Bradley's classification [43]. Based on morphological characteristics, phage ArV2 belongs to the family *Siphoviridae* [44] and is characterized by an isometric head (diameter, 62.8±4.9 nm; *n* = 30) and an apparently non-contractile, flexible tail (194.4±9.6 nm in length and 11.8±1.1 nm in width; *n* = 30). A baseplate was observed, although its diameter was not clearly distinguishible from the contractile sheath. Tail fibers are not obviously visible but upon closer inspection, six baseplate-associated short tail fibers (7.5±1.2 nm in lenght) can be seen in the vicinity of the tail tips (Fig. 1D, Fig. 1E).

### The host range and physiological characteristics

In total, 40 bacterial strains (Table 1) were used to explore the host range of ArV2. With the exception of *Arthrobacter* sp. strain 68b, all of 2 *Acinetobacter* sp., 26 *Arthrobacter* sp., 1 *Citrobacter* sp., 1 *Enterobacter* sp., 1 *Erwinia* sp., 5 *Escherichia* sp., 2 *Pseudomonas* sp., 2 *Klebsiella* sp. and 1 *Salmonella* sp. strains were found to be resistant to ArV2. *Arthrobacter* sp. strain 68b was isolated from soil. Although a number of strains used in this study is not large, the diversity of bacteria tested allow presuming that the host range of ArV2 is limited to *Arthrobacter* only.

**Figure 2:**
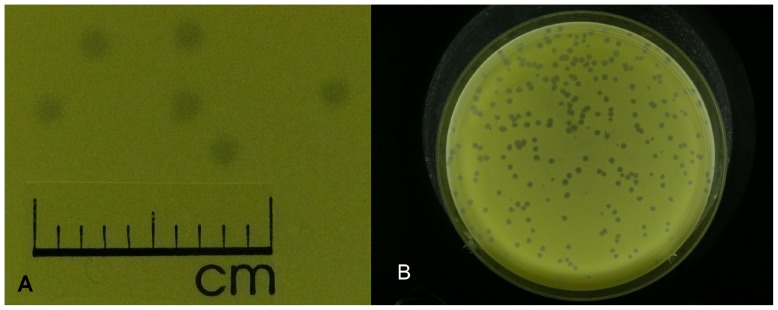
Plaques formed by bacteriophage ArV2 on a lawn of *Arthrobacter* sp. 68b.

To determine the optimal conditions for phage propagation, the efficiency of plating (e.o.p.) test was performed. The e.o.p. of ArV2 was examined in the temperature range of 15–37°C, and the test revealed that the phage has an optimum temperature for plating around 30°C (Fig. S1). After 48 h of incubation at an optimum temperature of 30°C, bacteriophage ArV2 formed clear plaques of 1.5±0.5 mm in diameter (Fig. 2). While ArV2 plaques can be visible after one day (24 h) of incubation at 30°C, an accurate evaluation of plaque morphology as well as the plaque enumeration is best performed after 36–48 h of incubation.

Attempts to obtain a one-step growth curve of ArV2 on strain 68b were unsuccessful because of the slow adsorption kinetics. Despite varying the inorganic environments (MgCl_2_ or CaCl_2_ solutions were added to the medium to reach the concentration of 10 mM) and temperature, only about 50% of the PFU adsorb in 5 min, and after 15 min as many as 25% of the original PFU remain unattached (data not shown). The slow adsorption kinetics of ArV2, taken together with extremely narrow host range, suggests that perhaps other bacteria, rather than *Arthrobacter* sp. 68b, is the real host of ArV2. On the other hand, these results may reflect the inherent nature of the phage, or the failure to provide the most appropriate environment for adsorption.

### Restriction analysis

**Figure 3:**
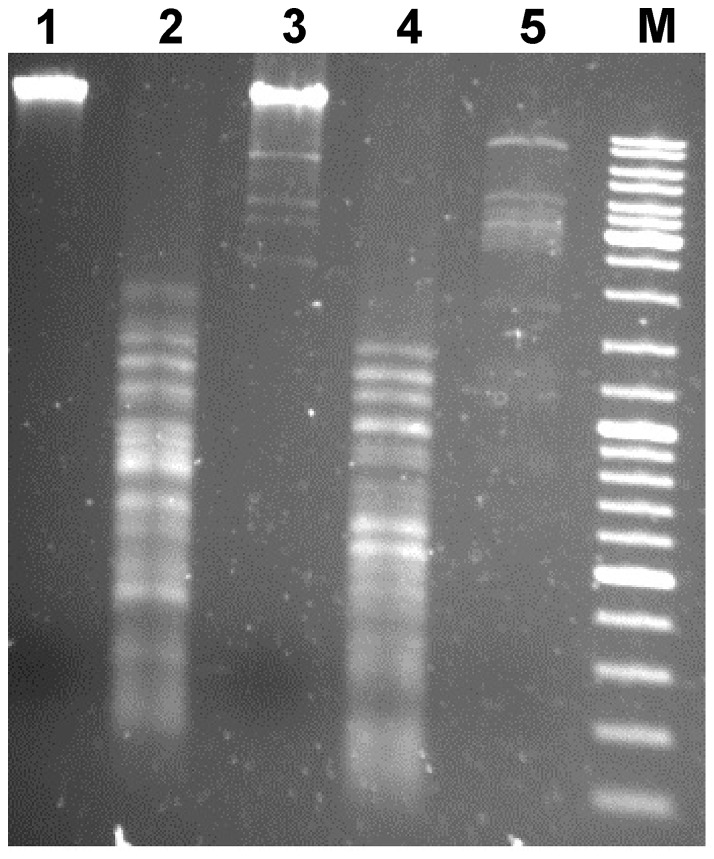
Restriction digestion patterns of phage ArV2 genomic DNA. Lanes: 1 – ArV2 gDNA (nonrestricted); 2 – EcoRII; 3 – NotI; 4 – MboI; 5 – BamHI; 6 – Marker GeneRulerTM DNA Ladder Mix (Thermo Fisher Scientific).

To protect the genomic DNA from restriction endonucleases, phages employ various strategies, including adenine and cytosine methylation as well as hydroxymethylation of cytosine (HMC) and subsequent glucosylation of HMC derivatives [45], [46]. Investigation of ArV2 DNA modifications was performed using EcoRII, NotI and MboI restriction endonucleases that do not cleave Dcm, CpG and Dam methylated DNA, respectively. The sensitivity of phage DNA to the restriction enzymes listed above suggests that the DNA of ArV2 possesses no significant amounts of bases with Dcm, CpG and Dam methylation (Fig. 3).

### Overview of the phage ArV2 genome

Phage ArV2 has a linear, double stranded DNA genome consisting of 37,372 bp with a G+C content of 62.73%, which is similar to that observed for *Arthrobacter* [9]. Since the restriction digestion profiles of phage ArV2 DNA matched *in silico* predictions of a linear DNA molecule, the locations of the terminase-generated ends (*cos* sites) were readily determined from a restriction map. By sequencing directly from the genome ends (data not shown), the *cos* sites of ArV2 were identified as 9-bp 3′ overhang sequences (5′-CCTCCGGCA-3′).

**Figure 4:**
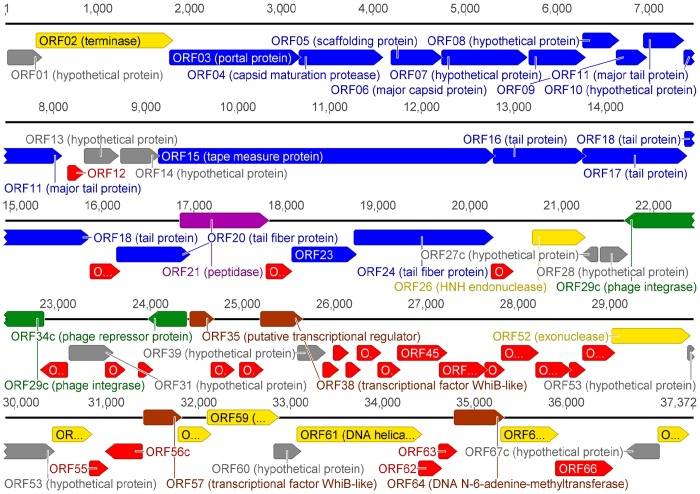
Functional genome map of bacteriophage ArV2. The coding capacity of the ArV2 genome is shown. Functions are assigned according to the characterized ORFs in NCBI database and/or MS/MS analysis. The colour code is as follows: yellow – DNA replication, recombination, repair and packaging; brown – transcription, translation, nucleotide metabolism; blue – structural proteins; purple – chaperones/assembly; green –lysogeny; grey – ORFs of unknown function; red – ArV2 specific ORFs that encode unique proteins that have no reliable identity to database entries.

The genome of ArV2 is close-packed: with an average ORF size of 535 bp, ∼96% of the genome is coding. It is not surprising since in tailed viruses protein-coding genes are generally tightly packed and typically occupy >90% of the genome [47]. The genome sequence analysis revealed that ArV2 has a total of 68 probable protein-encoding genes and no genes for tRNA (Fig. 4). While most of the ArV2 genes were found to initiate from ATG (37 out of 68 ORFs), 24 ORFs were found to initiate with GTG and 7 with TTG. A marked asymmetry in the distribution of the genes on the two phage ArV2 DNA strands was observed. In total, 62 (91%) ORFs were predicted to be transcribed from the same DNA strand, while 6 (9%) ORFs (most of these were detected in the lysogeny-related module) were found on the opposite strand.

The genome analysis revealed that 41% of the ArV2 genes (28 out of 68 ORFs) encode unique proteins that have no reliable identity (e-values >0.001) to database entries (Fig. 4). Among the ArV2 gene products with detectable homologs in other sequenced genomes, 34 were most similar to proteins from bacteria, such as *Arthrobacter*, *Microbacterium*, *Rhodococcus*, *Mobiluncus*, *Actinomyces*, *Lactococcus*, etc (Fig. S2A, Table S2). In addition, 24 of the aforementioned ArV2 ORFs showed amino acid sequence similarity, although to a lesser extent, to proteins from bacteriophages that infect *Mycobacterium*, *Propionibacterium*, *Streptomyces*, *Gordonia*, *Rhodococcus*, *Streptococcus* and other bacteria from the actinobacterial group (Fig. S2B). Only 6 ArV2 gene products showed sequence similarity exclusively to the homologous viral proteins (Fig. S2A, Table S2). Through the examination of homology search results, a putative function was assigned to 32 ArV2 ORFs that are distributed across several functional categories, such as head and tail morphogenesis, DNA packaging, lysogeny, replication and regulation (Fig. 4).

### DNA replication/recombination and modification enzymes

Several ArV2 gene products are potentially involved in nucleic acid replication or recombination processes. ORF54 was predicted to encode a ssDNA binding protein, which builds the scaffold of DNA replication and is reported to be essential both for DNA replication and for genetic recombination processes [48]. The protein encoded by ArV2 ORF61 shared 42% amino acid identity with the DNA helicase from *Streptomyces* sp. C, while the predicted product of ORF59 was found to be homologous to a DNA replication protein of *Lactobacillus* phage LF1 and showed weak homology to a DNA replication protein O from Stx2 converting phage vB_EcoP_24B. The DNA helicases are known to unwind the duplex DNA and are involved in replication, repair and recombination processes [49], [50], while the protein O has been reported to be necessary for the initiation of bacteriophage DNA replication [51]. Interestingly, although most of the phages encode their own conserved DNA polymerases [52], there is no DNA polymerase gene identified in the ArV2 genome suggesting that this phage takes advantage of host replication machinery.

Other ArV2 ORFs possibly involved in DNA recombination include a RecE exonuclease VIII (ORF52), a Holliday junction resolvase RusA [53] encoded by ORF65, two free-standing HNH endonucleases (ORF68 and ORF26) and one GIY-YIG nuclease (ORF058). Phage RecE 5′–3′ exonucleases act on dsDNA by production of a protruding 3′ ssDNA for strand annealing or invasion in homologous recombination [54], [55], while homing endonucleases are a distinctive class of site-specific DNA endonucleases that promote the lateral transfer of their own coding region and flanking DNA between genomes by a recombination-dependent process termed homing [56], [57].

Based on the amino acid sequence similarity, only one gene product of ArV2 was assigned as a protein involved in the DNA modification process. The derived protein product of ORF64 showed 58% amino acid sequence identity to the DNA-(*N*6-adenine)-methyltransferase from *Burkholderia* phage Bcep176 and was found to have a conserved Dam domain. According to the literature, adenine-specific DNA methyltransferases are often involved in chromosomal site-specific DNA modification systems, and it has been speculated that some bacteriophages express their own methylases to protect the genomic DNA against restriction enzymes of the host [58], [59]. However, as it was mentioned above, the restriction analysis revealed that the DNA of ArV2 seems to possess no significant amounts of bases with Dcm, CpG or Dam methylation (Fig. 3) suggesting that the predicted DNA-(*N*6-adenine)-methyltransferase from ArV2 is either not functional or modifies just a negligible amounts of nucleotides.

### DNA packaging

Packaging of phage genomes into empty procapsids is powered by the terminase holoenzyme that is generally composed of two subunits, large and small. In tailed phages, the small subunit of the terminase is responsible for specific DNA binding and holoenzyme formation, while the large subunit of the terminase mediates cleavage of the phage DNA into genome size units to be packaged into the prohead [60]. Bioinformatics analysis revealed that the large terminase subunit of ArV2 may be encoded by ORF02, as it shares 46% amino acid sequence identity with the terminase large subunit from *Mycobacterium* phage Validus, while the gene for a small terminase subunit seems to absent in ArV2. Nevertheless, in tailed phages, the gene encoding terminase small subunit is generally located upstream of the terminase large subunit gene, and both terminase genes are transcribed together in an operon-like structure [61], [62]. In ArV2, the gene position of ORF01 is upstream of the terminase large subunit gene suggesting that the product derived from this ORF is a potential candidate to be a novel small terminase. However, experimental data are needed to confirm this suggestion.

Besides terminase, another phage protein involved in packaging is the head-tail connector (or portal protein), which is the key functional component of the capsid for DNA packaging and is involved in the signaling for packaging termination [63], [64]. Comparative sequence analysis revealed that the portal protein of ArV2 is most likely encoded by the gene ArV2 ORF03, since it exhibited a moderate amino acid sequence identity (33%) to the portal protein from *Mycobacterium* phage BPs.

### Structural proteins

As was observed in other siphoviruses [61], [65], the packaging module in ArV2 is followed by a large genome cluster (∼18 kb) that contains genes encoding structural components of the virion. Bioinformatics analysis allowed us to identify 9 structural proteins of ArV2, including those coding for head (ORF03, ORF06), tail (ORF11, ORF15–ORF18), tail fiber (ORF20, ORF24), as well as a capsid maturation protease (ORF04) and a scaffolding protein (ORF05).

Reversed-phase nano-liquid chromatography directly coupled with LC-MS/MS analysis of the structural ArV2 proteins separated by SDS PAGE, and filter-aided protein sample preparation (FASP) led to the experimental identification of 14 virion proteins (Table 2), including 9 that were predicted by bioinformatics approaches as well as 5 proteins, which either have no detectable homology to any entries in the public databases (ORF09, ORF23) or are similar to hypothetical proteins from other phages (ORF07) and bacteria (ORF08, ORF10). Unexpectedly, a putative capsid maturation protease encoded by ArV2 ORF04, which has poor (23% identity) but significant amino acid sequence similarity to a capsid maturation protease of *Mycobacterium* phage Job42, was identified by MS/MS. In most phages, such proteins as morphogenesis-associated proteases are thought to be lost from the structure during capsid maturation [66], however, in the case of certain bacterial viruses, such as the coliphage P2 or mycobacteriophage Marvin, the protease is retained in mature virions [66], [67].

**Table 2:** Mass spectrometry analysis of phage ArV2 proteins.

Gene	Putative Function	MW (KDa)	Peptide count	Sequence coverage (%)
ORF15*	tape measure protein	127.953	74	35.2
ORF24*	tail fiber protein	52.293	11	38.0
ORF03*	phage portal protein	50.447	60	79.3
ORF17*	tail protein	40.386	42	70.1
ORF16*	tail protein	35.644	26	58.3
ORF18*	tail protein	33.604	39	84.9
ORF04^f^	capsid maturation protease	31.057	19	63.3
ORF06*	major capsid protein	30.937	21	78.9
ORF11*	major tail protein	23.801	72	95.9
ORF23^u^	hypothetical protein	20.621	16	95.4
ORF07	hypothetical protein	14.566	13	91.2
ORF10	hypothetical protein	14.563	9	70.4
ORF08	hypothetical protein	12.709	7	78.8
ORF09* ^f, u^	hypothetical protein	10.230	10	76.2

* – putative structural ArV2 proteins identified by bioinformatics approaches;

f – proteins detected using the FASP method only;

u – ArV2-specific proteins that have no reliable identity to database entries.

Proteins were identified as a result of whole-phage shotgun analysis (WSA) using LC MS/MS analysis and Mascot search against an in-house database.

**Figure 5:**
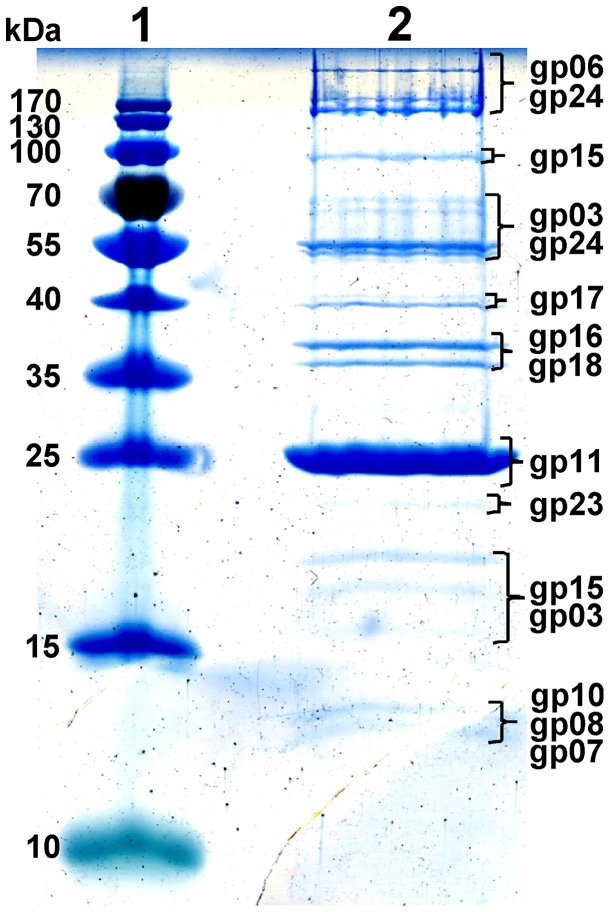
SDS-PAGE of virion proteins of ArV2. Lanes: 1– molecular mass marker Page RulerTM prestained Protein Ladder Plus (Thermofisher), 2– phage ArV2 structural proteins. Relative migrations of MW marker proteins are indicated on the left. Proteins identified by MS/MS are indicated on the right.

The most abundant protein in the ArV2 virion (Fig. 5) was the major tail subunit (gp11) that shared 29% identity with the putative major tail protein from *Corynebacterium* phage P1201. In contrast to what has been observed in other tailed viruses, the predicted major capsid protein (gp06) of ArV2 was not the most abundant protein in the virion. The ArV2 ORF06 was predicted to encode a 30.9 kDa protein (298 residues) that shares 51% identity with the major capsid protein from *Mycobacterium* phage Severus. As seen from Fig. 5, gp06 was found to migrate with a molecular mass much larger than predicted, suggesting the covalent self-linking of the capsid proteins, a structural feature observed with other phage capsid proteins [68], [62], [69]. The cross-linking of the capsid proteins may explain a relatively low amount of gp06 detected by LC-MS/MS, presumably because cross-linking leads to the failure of the protein to enter the gel. The largest gene product (1209 aa) identified in the ArV2 genome was the tape measure protein (gp15) that showed 35% identity to the tape measure protein (TMP) from *Rhodococcus* phage ReqiPine5. The TMP usually functions as a template for measuring tail length during tail assembly [70]. As it can be seen in Fig. 5, gp15 was found to migrate with a molecular mass slightly lower than predicted (100 kDa vs 128 kDa). According to the literature, it is quite common for TMPs to be proteolytically processed during phage assembly [71], [72], [73]. Hence, the size reduction of ArV2 gp15 observed by SDS-PAGE suggests that this protein may be subjected to proteolytic cleavage.

At least five more tail proteins (gp16–18, gp20 and gp24) were detected in the virion of ArV2. The products of ORF16, ORF17 and ORF18 shared 30%, 33% and 32% amino acid sequence identity with putative minor tail proteins gp15, gp16 and gp17 from *Propionibacterium* phage P100_1, respectively. Meanwhile gp20 and gp24 of ArV2 were similar to the gp7 from *Mycobacterium* phage Trixie (47% aa identity) and to the hypothetical protein from *Rhodococcus opacus* B4 (32% aa identity), respectively.

Based on the results of proteomics analysis, the proteins encoded by ORF03, ORF05 and ORF21 were also present in ArV2 virion. A comparative sequence analysis revealed that ORF03 coded for portal protein (see above), meanwhile the product encoded by ORF05, which was located immediately upstream of the predicted major capsid protein (ORF06), was identified as the scaffolding protein and exhibited a moderate sequence identity (38%) to the gp6 from *Mycobacterium* phage Ramsey. Finally, the protein of ORF21 was identified as peptidase and shared 44% amino acid sequence identity with hypothetical protein from *Arthrobacter* sp. AK-YN10.

As was the case with other bacterial viruses [33], two structural proteins of ArV2 (gp05, gp20) predicted by bioinformatics approaches were not detectable by mass spectrometry, suggesting the incompatibility of such peptides with sample preparation procedures or/and their low abundance in virions.

### Lysis, phage-host interactions

Bacteriophage release usually involves a two-gene lysis cassette composed of a holin and an endolysin. The holin creates pores in the inner or cytoplasmic membrane permitting the endolysin to access the peptidoglycan layer in the periplasm resulting in cell lysis and release of progeny viruses [74], [75], [76]. However, while phage ArV2 is obviously capable of lysing *Arthrobacter* sp 68b cells, no characterized holins or endolysins have detectable homologues in the genome of ArV2.

A screen of virion constituent proteins of diverse tailed phages revealed that many different phage particles carry peptidoglycan hydrolytic activities, such as the peptidoglycan-degrading domains located in the TMPs of phage T5 and mycobacteriophage TM4 or a M23 family peptidase motif found in the Tal protein of *L. lactis* phage Tuc2009 [77], [78] Bacteriophage ArV2 harbours two virion proteins (gp15 and gp21) that were found to contain a conserved catalytic peptidase_M23 domain. Members of peptidase family M23 are zinc metallopeptidases with Gly-Gly endopeptidase activity and many of them, such as lysostaphin, have specific hydrolytic activity towards peptidoglycan [79], [77].

### Lysogeny

The lysogeny module of ArV2 was found downstream of the structural module. Unsurprisingly, since lysogeny-related genes usually are in the opposite orientation on the complementary strand relative to the other functional modules [80], two of the lysogeny-related genes of ArV2, namely integrase (ORF29c) and phage repressor (ORF34c), were found on the complementary strand (Fig. 4). The predicted phage repressor protein, which is critical to preservation of lysogeny [81], was encoded by ORF34c and shared 42% amino acid sequence identity with cI-like repressor from *Streptococcus* phage Sfi21. The product of ORF29c, which showed consistent homology to phage integrases (Fig. S3), was predicted to belong to the integrase family of tyrosine recombinases (Int family) [82]. Phage integrases are site-specific recombinases that mediate recombination between the phage genome and the bacterial chromosome [83]. According to the literature, Int integrases, such as lambda integrase, utilize a catalytic tyrosine to mediate strand cleavage and require cofactors encoded by the phage or the host bacteria [83]. Nevertheless, no phage encoded cofactors were found in the genome of the phage ArV2, suggesting that this phage either utilizes host-encoded cofactors or harbors peptides with negligible identity to known Cro-like proteins. On the other hand, there is also a possibility that ArV2 is a lysogenic phage that begins the lytic cycle spontaneously.

To determine whether lysogens could be recovered from ArV2 infections, cells from a spot where ArV2 particles had infected a lawn of *Arthrobacter* sp. 68b were recovered and grown on solid media. Bacterial growth was observed, and two independent colonies were restreaked twice more and then patched onto *Arthrobacter* sp. 68b lawns to test for phage release; none of the colonies recovered showed phage release (data not shown). Thus, although lysogeny-related genes are typically observed in temperate phages [84], [85], there is no evidence that ArV2 is capable of lysogenizing *Arthrobacter* sp. 68b.

### Phylogenetic relatedness

**Figure 6:**
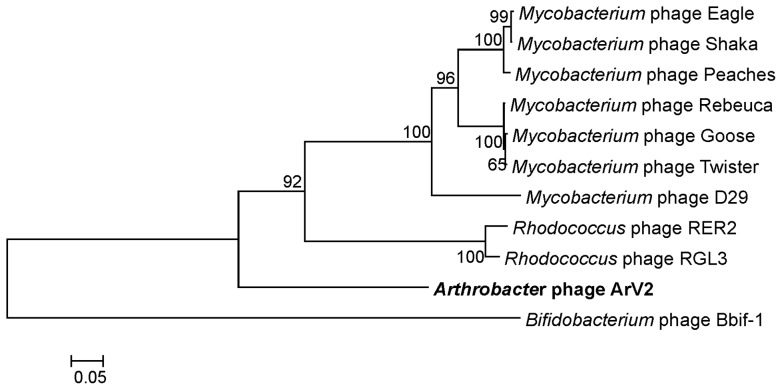
Neighbor-joining tree analysis based on the alignment of the amino acid sequences of the major capsid proteins from various tailed viruses. The bootstrap values are indicated.

**Figure 7:**
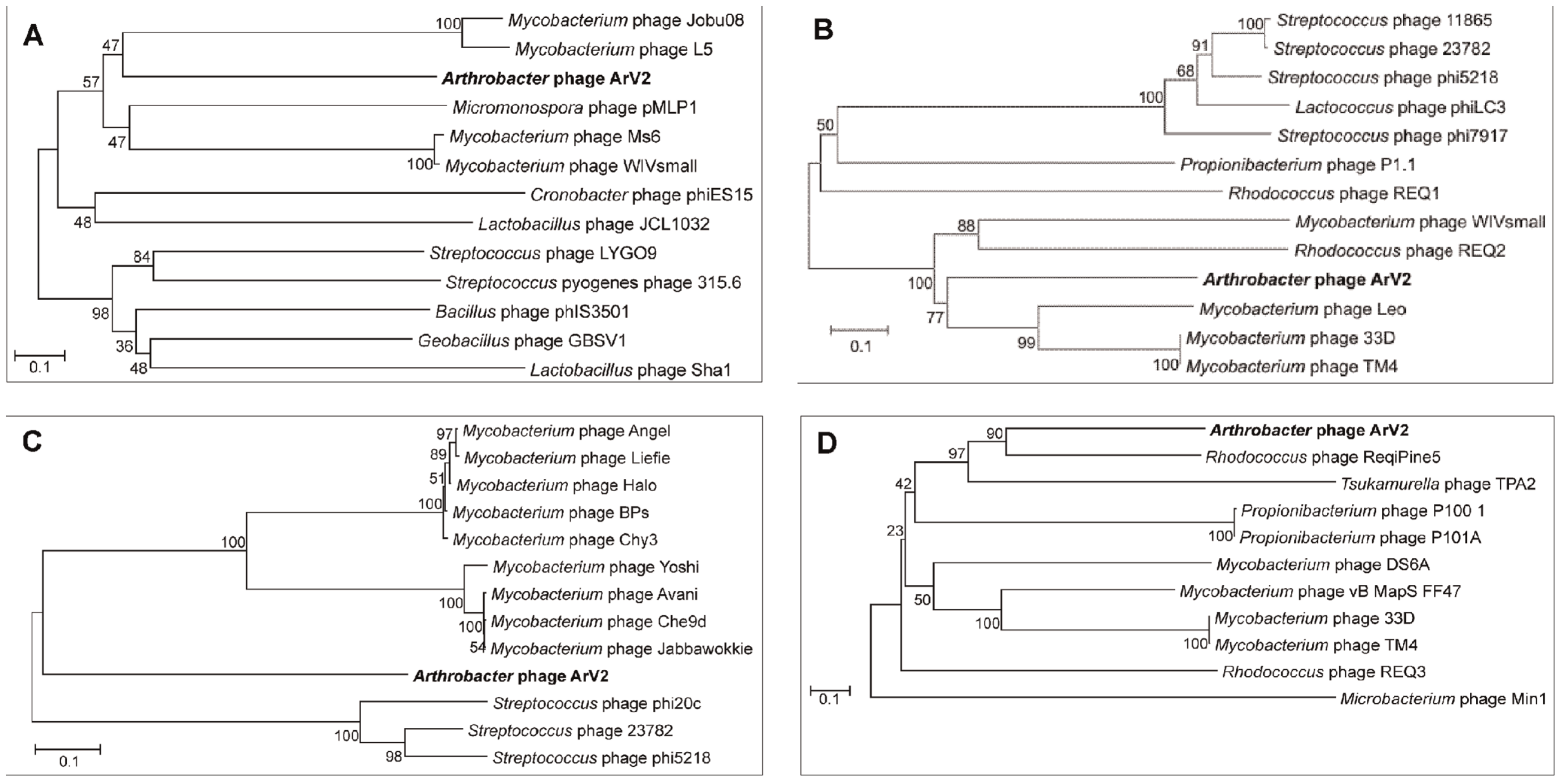
Phylogenetic analysis. Relationships of (A) integrase; (B) terminase; (C) portal protein; (D) tail tape measure protein across diverse phage types. The bootstrap values are indicated.

As observed with other bacterial viruses, gene content and genetic identity are highly heterogenous between phages and, more often than not, prevent the application of traditional phylogenetic methods using whole genome sequences [86]. A comparative sequence analysis revealed that the genome of *Arthrobacter* sp. infecting bacteriophage ArV2 shares no identity with any other sequenced genome. Moreover, at the amino acid sequence level, the majority of functionally annotated proteins of ArV2 are much more closely related to different bacterial proteins than to those from other sequenced viral genomes. Hence, the single-gene comparison approach was undertaken, and phylogenetic analysis of the major capsid protein (Fig. 6) as well as four other ubiquitous proteins, including terminase, integrase, tail tape measure protein and portal protein, was carried out to better understand the evolutionary relationships between ArV2 and other tailed viruses (Fig. 7). All five phylogenetic trees showed that ArV2 is phylogenetically distant from other phages and, most likely, represents an evolutionarily distinct branch within the family *Siphoviridae*.

## Conclusions

To our knowledge, this study represents the first complete genome sequence and genetic characterization of an *Arthrobacter* sp. infecting bacteriophage. Bioinformatics analysis revealed that the genome of ArV2 is significantly divergent from the siphovirus genomes sequenced to date, so much so that even structural proteins required experimental validation to annotate while a number of functionally important proteins (e.g. DNA polymerase, holin and endolysin) remained undetected. Undoubtedly, further genome sequencing and bioinformatic analysis should be performed to overcome the lack of genome annotation information and to draw a more detailed view of this particular group of bacterial viruses. However, the results of this study may provide new insights that deepen our understanding of *Arthrobacter* phage genetics and phage-host interactions in dynamic ecosystems, such as soil.

## Supporting Information

Figure S1 **Effect of temperature on the efficiency of plating of phage ArV2.**
(TIFF)

Figure S2 **Taxonomy of the best BlastP hits of ArV2 ORFs.** (A). The taxonomic distribution of homologues in phages that were grouped according to host specificity (B).
(TIFF)

Figure S3 **Sequence alignment of the Int family integrase from** ***Arthrobacter*** **phage Arv2 and other organisms.** Conserved amino acid positions demonstrated by Nunes-Düby et al. [82] are shaded.
(TIFF)

Table S1 **The diversity of** ***Arthrobacter*** **bacteriophages described to date.** S – *Siphoviridae*, P – *Podoviridae*, nd – not determined.
(DOC)

Table S2 **ArV2 ORFs with homologues in other viruses or cellular organisms.**
(DOC)

## References

[1] Cacciari I, Lippi D (1987) *Arthrobacters*: successful arid soil bacteria. A review. Arid Soil Res Rehabil 1: 1–30.

[2] Jones D, Keddie RM (2006) The genus *Arthrobacter*. In: Dworkin M, Falkow S, Rosenberg E, Schleifer KH, Stackebrandt E, editors. The Prokaryotes. Springer, New York, USA. 945–960.

[3] Hagedorn C, Holt JG (1975) A nutritional and taxonomic survey of *Arthrobacter* soil isolates. Can J Microbiol 21: 353–361. 1116046 10.1139/m75-050

[4] Roh SW, Sung Y, Nam YD, Chang HW, Kim KH, et al (2008) *Arthrobacter soli* sp. nov., a novel bacterium isolated from wastewater reservoir sediment. J Microbiol 46: 40–44. 18337691 10.1007/s12275-007-0239-8

[5] Huang Y, Zhao N, He L, Wang L, Liu Z, et al (2005) *Arthrobacter scleromae* sp. nov. isolated from human clinical specimens. J Clin Microbiol 43: 1451–1455. 15750131 10.1128/JCM.43.3.1451-1455.2005 PMC1081264

[6] Collins MD, Hoyles L, Foster G, Falsen E, Weiss N (2002) *Arthrobacter nasiphocae* sp. nov., from the common seal (*Phoca vitulina*). In J Syst Evol Microbiol 52: 569–571. 10.1099/00207713-52-2-569 11931170

[7] Scheublin TR, Leveau JH (2013) Isolation of *Arthrobacter* species from the phyllosphere and demonstration of their epiphytic fitness. Microbiologyopen 2: 205–213. 23355506 10.1002/mbo3.59 PMC3584225

[8] Heyrman J, Verbeeren J, Schumann P, Swings J, Paul DV (2005) Six novel *Arthrobacter* species isolated from deteriorated mural paintings. Int J Syst Evol Microbiol 55: 1457–1464. 16014466 10.1099/ijs.0.63358-0

[9] Monnet C, Loux V, Gibrat JF, Spinnler E, Barbe V, et al (2010) The *Arthrobacter arilaitensis* Re117 genome sequence reveals its genetic adaptation to the surface of cheese. PLoS ONE 5: e15489. 21124797 10.1371/journal.pone.0015489 PMC2991359

[10] Li Y, Kawamura Y, Fujiwara N, Naka T, Liu H, et al (2004) *Rothia aeria* sp. nov., *Rhodococcus baikonurensis* sp. nov. and *Arthrobacter russicus* sp. nov., isolated from air in the Russian space laboratory Mir. Int J Syst Evol Microbiol 54: 827–835. 15143031 10.1099/ijs.0.02828-0

[11] Pindi PK, Manorama R, Begum Z, Shivaji S (2010) *Arthrobacter antarcticus* sp. nov., isolated from an Antarctic marine sediment. Int J Syst Evol Microbiol 60: 2263–2266. 19783612 10.1099/ijs.0.012989-0

[12] Hanbo Z, Changqun D, Qiyong S, Weimin R, Tao S, et al (2004) Genetic and physiological diversity of phylogenetically and geographically distinct groups of *Arthrobacter* isolated from lead-zinc mine tailings. FEMS Microbiol Ecol 49: 333–341. 19712425 10.1016/j.femsec.2004.04.009

[13] Fredrickson JK, Zachara JM, Balkwill DL, Kennedy D, Li SM, et al (2004) Geomicrobiology of high-level nuclear waste-contaminated vadose sediments at the Hanford site, Washington state. Appl Environ Microbiol 70: 4230–4241. 15240306 10.1128/AEM.70.7.4230-4241.2004 PMC444790

[14] Kutanovas S, Rutkienė R, Urbelis G, Tauraitė D, Stankevičiūtė J, et al (2013) Bioconversion of methylpyrazines and pyridines using novel pyrazines-degrading microorganisms. Chemija 24: 67–73.

[15] Marshall SJ, White GF (2001) Complete denitration of nitroglycerin by bacteria isolated from a washwater soakaway. Appl Environ Microbiol 67: 2622–2626. 11375172 10.1128/AEM.67.6.2622-2626.2001 PMC92916

[16] Robinson JB, Corke CT (1959) Preliminary studies on the distribution of actinophages in soil. Can J Microbiol 5: 479–484. 14437962 10.1139/m59-059

[17] Conn HJ, Bottcher EJ, Randall C (1945) The value of bacteriophage in classifying certain soil bacteria. J Bacteriol 49: 359–373. 16560929 10.1128/jb.49.4.359-373.1945 PMC374053

[18] Brown DR, Holt JG, Pattee PA (1978) Isolation and characterization of *Arthrobacter* bacteriophages and their application to typing of soil *Arthrobacter* . Appl Environ Microbiol 35: 185–191. 74980 10.1128/aem.35.1.185-191.1978 PMC242800

[19] Gillespie DC (1960) Isolation of bacteriophage for *Arthrobacter globiformis* . Can J Microbiol 6: 477–478.

[20] Schippers-Lammertse AF, Mulisers AO, Klatser-Oedekerk KB (1963) *Arthrobacter polychromogenes* nov. spec., its pigment, and a bacteriophage of this species. Antonie van Leeuwenhoek 29: 1–15. 10.1007/BF02046034 14024588

[21] Casida LE Jr, Liu KC (1974) *Arthrobacter globiformis* and its bacteriophage in soil. Appl Microbiol 28: 951–959. 16350007 10.1128/am.28.6.951-959.1974 PMC186862

[22] Ostle AG, Holt JG (1979) Elution and inactivation of bacteriophages on soil and cation exchange resin. Appl Environ Microbiol 38: 59–65. 16345416 10.1128/aem.38.1.59-65.1979 PMC243435

[23] Germida JJ, Casida LE Jr (1981) Isolation of *Arthrobacter* bacteriophage from soil. Appl Environ Microbiol 41: 1389–1393. 16345792 10.1128/aem.41.6.1389-1393.1981 PMC243928

[24] Trautwetter A, Blanco C (1988) Isolation and preliminary characterization of twenty bacteriophages infecting either *Brevibacterium* or *Arthrobacter* strains. Appl Environ Microbiol 54: 1466–1471. 16347657 10.1128/aem.54.6.1466-1471.1988 PMC202681

[25] Daems WT (1963) A preliminary report on the fine structure of a bacteriophage of *Arthrobacter polychromogenes* . Antonie Van Leeuwenhoek 29: 16–21. 14024588 10.1007/BF02046034

[26] Einck KH, Pattee PA, Holt JG, Hagedorn C, Miller JA, et al (1973) Isolation and characterization of a bacteriophage of *Arthrobacter globiformis* . J Virol 12: 1031–1033. 4128824 10.1128/jvi.12.5.1031-1033.1973 PMC356733

[27] Le Marrec C, Michotey V, Blanco C, Trautwetter A (1994) ΦAAU2, a temperate bacteriophage for *Arthrobacter aureus*, whose integrative functions work in other corynebacteria. Microbiology 140: 3071–3077.

[28] Ackermann HW, Prangishvili D (2012) Prokaryote viruses studied by electron microscopy Arch Virol. 157: 1843–1849. 10.1007/s00705-012-1383-y 22752841

[29] Stanislauskienė R, Rudenkov M, Karvelis L, Gasparavičiūtė R, Meškienė R, et al (2011) Analysis of phthalate degradation operon from *Arthrobacter* sp. 68b. Biologija 57: 45–54.

[30] Semėnaitė R, Gasparavičiūtė R, Duran R, Precigou S, Marcinkevičienė L, et al (2003) Genetic diversity of 2-hydroxypyridine-degrading soil bacteria. *Biologija* 2: 27–29.

[31] Stanislauskiene R, Gasparaviciute R, Vaitekunas J, Meskiene R, Rutkiene R, et al (2012) Construction of *Escherichia coli*-*Arthrobacter*-*Rhodococcus* shuttle vectors based on a cryptic plasmid from *Arthrobacter rhombi* and investigation of their application for functional screening. FEMS Microbiol Lett 327: 78–86. 22098420 10.1111/j.1574-6968.2011.02462.x

[32] Gasparavičiūtė R, Kropa A, Meškys R (2006) A new *Arthrobacter* strain utilizing 4-hydroxypyridine. Biologija 4: 41–45.

[33] Šimoliūnas E, Kaliniene L, Truncaitė L, Zajančkauskaitė A, Staniulis J, et al (2013) *Klebsiella* phage vB_KleM-RaK2 – a giant singleton virus of the family *Myoviridae* . PLoS ONE 8: e60717. 23593293 10.1371/journal.pone.0060717 PMC3622015

[34] Adams MH (1959) Bacteriophages. Interscience Publishers, New York, USA.

[35] Sambrook J, Russel D (2001) Molecular cloning: a laboratory manual. Cold Spring Harbor Laboratory Press, Cold Spring Harbor, New York.

[36] Kropinski AM (2009) Measurement of the rate of attachment of bacteriophage to cells. In: Clokie MRJ, Kropinski AM, editors. Bacteriophages: methods and protocols. Humana Press, New York, USA. 151–155. 10.1007/978-1-60327-164-6_15 19066819

[37] Kaliniene L, Klausa V, Truncaite L (2010) Low-temperature T4-like coliphages vB_EcoM-VR5, vB_EcoM-VR7 and vB_EcoM-VR20. Arch Virol 155: 871–880. 20361343 10.1007/s00705-010-0656-6

[38] Carlson K, Miller E (1994) Experiments in T4 genetics. In: Karam JD, editor. Bacteriophage T4. ASM Press, Washington DC, USA. 419–483.

[39] Tamura K, Peterson D, Peterson N, Stecher G, Nei M, et al (2011) MEGA5: molecular evolutionary genetics analysis using maximum likelihood, evolutionary distance, and maximum parsimony methods. Mol Biol Evol 28: 2731–2739. 21546353 10.1093/molbev/msr121 PMC3203626

[40] Laemmli UK (1970) Cleavage of structural proteins during the assembly of the head of bacteriophage T4. Nature 227: 680–685. 5432063 10.1038/227680a0

[41] Wisniewski JR, Zougman A, Nagaraj N, Mann M (2009) Universal sample preparation method for proteome analysis. Nat Methods 6: 359–362. 19377485 10.1038/nmeth.1322

[42] Hellman U, Wernstedt C, Gonez J, Heldin CH (1995) Improvement of an “in-gel” digestion procedure for the micropreparation of internal protein fragments for amino acid sequencing. Anal Biochem 224: 451–5. 7710111 10.1006/abio.1995.1070

[43] Bradley DE (1967) Ultrastructure of bacteriophages and bacteriocins. Bacteriol Rev 31: 230–314. 4865539 10.1128/br.31.4.230-314.1967 PMC408286

[44] Ackermann HW (2001) Frequency of morphological phage descriptions in the year 2000. Arch Virol 146: 843–857. 11448025 10.1007/s007050170120

[45] Warren RA (1980) Modified bases in bacteriophage DNAs. Ann Rev Microbiol 34: 137–158. 7002022 10.1146/annurev.mi.34.100180.001033

[46] Miller ES, Kutter E, Mosig G, Arisaka F, Kunisawa T, et al (2003) Bacteriophage T4 genome. Microbiol Mol Biol Rev 67: 86–156. 12626685 10.1128/MMBR.67.1.86-156.2003 PMC150520

[47] Hendrix RW (2002) Bacteriophages: evolution of the majority. Theor Popul Biol 61: 471–480. 12167366 10.1006/tpbi.2002.1590

[48] Werten S (2013) Identification of the ssDNA-binding protein of bacteriophage T5: Implications for T5 replication. Bacteriophage 3: e27304. 24482743 10.4161/bact.27304 PMC3897522

[49] Lionnet T, Spiering MM, Benkovic SJ, Bensimon D, Croquette V (2007) Real-time observation of bacteriophage T4 gp41 helicase reveals an unwinding mechanism. Proc Natl Acad Sci USA 104: 19790–19795. 18077411 10.1073/pnas.0709793104 PMC2148377

[50] Tuteja N, Tuteja R (2004) Prokaryotic and eukaryotic DNA helicases. Essential molecular motor proteins for cellular machinery. Eur J Biochem 271: 1835–1848. 15128294 10.1111/j.1432-1033.2004.04093.x PMC7164108

[51] Zylicz M, Gorska I, Taylor K, Georgopoulos C (1984) Bacteriophage lambda replication proteins: formation of a mixed oligomer and binding to the origin of lambda DNA. Mol Gen Genet 196: 401–406. 6239082 10.1007/BF00436186

[52] Li P, Chen B, Song Z, Song Y, Yang Y, et al (2012) Bioinformatic analysis of the *Acinetobacter baumannii* phage AB1 genome. Gene 507: 125–134. 22868206 10.1016/j.gene.2012.07.029

[53] Sharples GJ, Chan SN, Mahdi AA, Whitby MC, Lloyd RG (1994) Processing of intermediates in recombination and DNA repair: identification of a new endonuclease that specifically cleaves Holliday junctions. EMBO Journal 13: 6133–6142. 7813450 10.1002/j.1460-2075.1994.tb06960.x PMC395593

[54] Kolodner R, Hall SD, Luisi-DeLuca C (1994) Homologous pairing proteins encoded by the *Escherichia coli* recE and recT genes. Mol Microbiol 11: 23–30. 8145642 10.1111/j.1365-2958.1994.tb00286.x

[55] Stahl MM, Thomason L, Poteete AR, Tarkowski T, Kuzminow A, et al (1997) Annealing vs. invasion in phage lambda recombination. Genetics 147: 961–977. 9383045 10.1093/genetics/147.3.961 PMC1208271

[56] Belfort M, Derbyshire V, Cousineau B, Lambowitz A (2002) Mobile introns: pathways and proteins. In: Craig N, Craigie R, Gellert M, Lambowitz A, editors. Mobile DNA II. Am Soc Microbiol, Washington, DC. 761–783.

[57] Friedrich NC, Torrents E, Gibb EA, Sahlin M, Sjöberg BM, et al (2007) Insertion of a homing endonuclease creates a genes-in-pieces ribonucleotide reductase that retains function. Proc Natl Acad Sci U S A 104: 6176–6181. 17395719 10.1073/pnas.0609915104 PMC1851037

[58] Marinus MG (1996) Methylation of DNA. In: Neidhardt FC, Curtiss III R, Ingraham JL, Lin ECC, Low KB, et al, editors. editors. *Escherichia coli* and *Salmonella*: cellular and molecular biology 2nd ed., vol. 1. ASM Press, Washington, D. C: 697–702.

[59] Lu Z, Altermann E, Breidt F, Kozyavkin S (2010) Sequence analysis of *Leuconostoc mesenteroides* bacteriophage Phi1-A4 isolated from an industrial vegetable fermentation. Appl Environ Microbiol 76: 1955–1966. 20118355 10.1128/AEM.02126-09 PMC2838018

[60] Jardine PJ, Anderson DL (2006) DNA packaging in double-stranded DNA phages. In: Calendar R, Abedon ST, editors. The bacteriophages 2nd edn. Oxford University Press, Oxford, N.Y. 49–65.

[61] Brüssow H, Desiere F (2001) Comparative phage genomics and the evolution of *Siphovirdae*: insights from dairy phages. Mol Microbiol 39: 213–222. 11136444 10.1046/j.1365-2958.2001.02228.x

[62] Petrovski S, Dyson ZA, Seviour RJ, Tillett D (2012) Small but sufficient: the *Rhodococcus* phage RRH1 has the smallest known *Siphoviridae* genome at 14.2 kilobases J Virol. 86: 358–363. 10.1128/JVI.05460-11 PMC3255915 22013058

[63] Tavares P, Zinn-Justin S, Orlova EV (2012) Genome gating in tailed bacteriophage capsids. Adv Exp Med Biol 726: 585–600. 22297531 10.1007/978-1-4614-0980-9_25

[64] Rao VB, Feiss M (2008) The bacteriophage DNA packaging motor. Annu Rev Genet 42: 647–681. 18687036 10.1146/annurev.genet.42.110807.091545

[65] Desiere F, Lucchini S, Canchaya C, Ventura M, Brüssow H (2002) Comparative genomics of phages and prophages in lactic acid bacteria. Antonie Van Leeuwenhoek 82: 73–91. 12369206

[66] Mageeney C, Pope WH, Harrison M, Moran D, Cross T, et al (2012) *Mycobacteriophage* Marvin: a new singleton phage with an unusual genome organization. J Virol 86: 4762–4775. 22357284 10.1128/JVI.00075-12 PMC3347389

[67] Chang JR, Poliakov A, Prevelige PE, Mobley JA, Dokland T (2008) Incorporation of scaffolding protein gpO in bacteriophages P2 and P4. Virology 370: 352–361. 17931675 10.1016/j.virol.2007.08.039 PMC2186210

[68] Petrovski S, Seviour RJ, Tillett D (2011) Characterization of the genome of the polyvalent lytic bacteriophage GTE2, which has potential for biocontrol of *Gordonia*-, *Rhodococcus*- and *Nocardia*-stabilized foams in activated sludge plants. Appl Environ Microbiol 77: 3923–3929. 21498753 10.1128/AEM.00025-11 PMC3131622

[69] Wikoff WR, Liljas L, Duda RL, Tsuruta H, Hendrix RW, et al (2000) Topologically linked protein rings in the bacteriophage HK97 capsid. Science 289: 2129–2133. 11000116 10.1126/science.289.5487.2129

[70] Katsura I, Hendrix RW (1984) Length determination in bacteriophage lambda tails. Cell 39: 691–698. 6096021 10.1016/0092-8674(84)90476-8

[71] Hendrix RW, Casjens SR (1974) Protein cleavage in bacteriophage lambda tail assembly. Virology 61: 156–159. 4415352 10.1016/0042-6822(74)90250-5

[72] Zimmer M, Sattelberger E, Inman RB, Calendar R, Loessner MJ (2003) Genome and proteome of *Listeria monocytogenes* phage PSA: an unusual case for programmed +1 translational frameshifting in structural protein synthesis. Mol Microbiol 50: 303–317. 14507382 10.1046/j.1365-2958.2003.03684.x

[73] Mc Grath S, Neve H, Seegers JF, Eijlander R, Vegge CS, et al (2006) Anatomy of a lactococcal phage tail. J Bacteriol 188: 3972–3982. 16707689 10.1128/JB.00024-06 PMC1482904

[74] Wang I, Smith DL, Young R (2000) Holins: the protein clocks of bacteriophage infections. Annu Rev Microbiol 54: 799–825. 11018145 10.1146/annurev.micro.54.1.799

[75] Roberts MD, Martin ML, Kropinski AM (2004) The genome and proteome of coliphage T1. Virology 318: 245–266. 14972552 10.1016/j.virol.2003.09.020

[76] Loessner MJ (2005) Bacteriophage endolysins-current state of research and applications. Curr Opin Microbiol 8: 480–487. 15979390 10.1016/j.mib.2005.06.002

[77] Fraser JS, Maxwell KL, Davidson AR (2007) Immunoglobulin-like domains on bacteriophage: weapons of modest damage? Curr Opin Microbiol 10: 382–387. 17765600 10.1016/j.mib.2007.05.018

[78] Davidson AR, Cardarelli L, Pell LG, Radford DR, Maxwell KL (2012) Long noncontractile tail machines of bacteriophages. Adv Exp Med Biol 726: 115–142. 22297512 10.1007/978-1-4614-0980-9_6

[79] Schindler CA, Schuhardt VT (1965) Purification and properties of lysostaphin – a lytic agent of *Staphylococcus aureus* . Biochem Biophys Acta 97: 242–250. 14292833 10.1016/0304-4165(65)90088-7

[80] Brüssow H, Canchaya C, Hardt WD (2004) Phages and the evolution of bacterial pathogens: from genomic rearrangements to lysogenic conversion. Microbiol Mol Biol Rev 68: 560–602. 15353570 10.1128/MMBR.68.3.560-602.2004 PMC515249

[81] Matthews BW, Ohlendorf DH, Anderson WF, Fisher RG, Takeda Y (1983) Cro repressor protein and its interaction with DNA. Cold Spring Harb Symp Quant Biol 47: 427–433. 6305561 10.1101/sqb.1983.047.01.050

[82] Nunes-Düby SE, Kwon HJ, Tirumalai RS, Ellenberger T, Landy A (1998) Similarities and differences among 105 members of the Int family of site-specific recombinases. Nucleic Acids Res 26: 391–406. 9421491 10.1093/nar/26.2.391 PMC147275

[83] Groth AC, Calos MP (2004) Phage integrases: biology and applications. J Mol Biol 335: 667–678. 14687564 10.1016/j.jmb.2003.09.082

[84] Canchaya C, Proux C, Fournous G, Bruttin A, Brüssow H (2003) Prophage genomics. Microbiol Mol Biol Rev 67: 238–276. 12794192 10.1128/MMBR.67.2.238-276.2003 PMC156470

[85] Brüssow H (2006) Prophage genomics. In: Calendar R, Abedon ST, editors. The Bacteriophages 2nd edn. Oxford University Press, Oxford, 17–25.

[86] Smith KC, Castro-Nallar E, Fisher JNB, Breakwell DP, Grose JH, et al (2013) Phage cluster relationships identified through single gene analysis. BMC Genomics 14: 410. 23777341 10.1186/1471-2164-14-410 PMC3698066

