# Supplementary figures and images for: Isolation and Characterization of vB_ArS-ArV2 – First Arthrobacter sp. Infecting Bacteriophage with Completely Sequenced Genome
Source: PLoS One. 2014 Oct 21;9(10):e111230. doi: 10.1371/journal.pone.0111230 (PMC4205034; doi:10.1371/journal.pone.0111230)

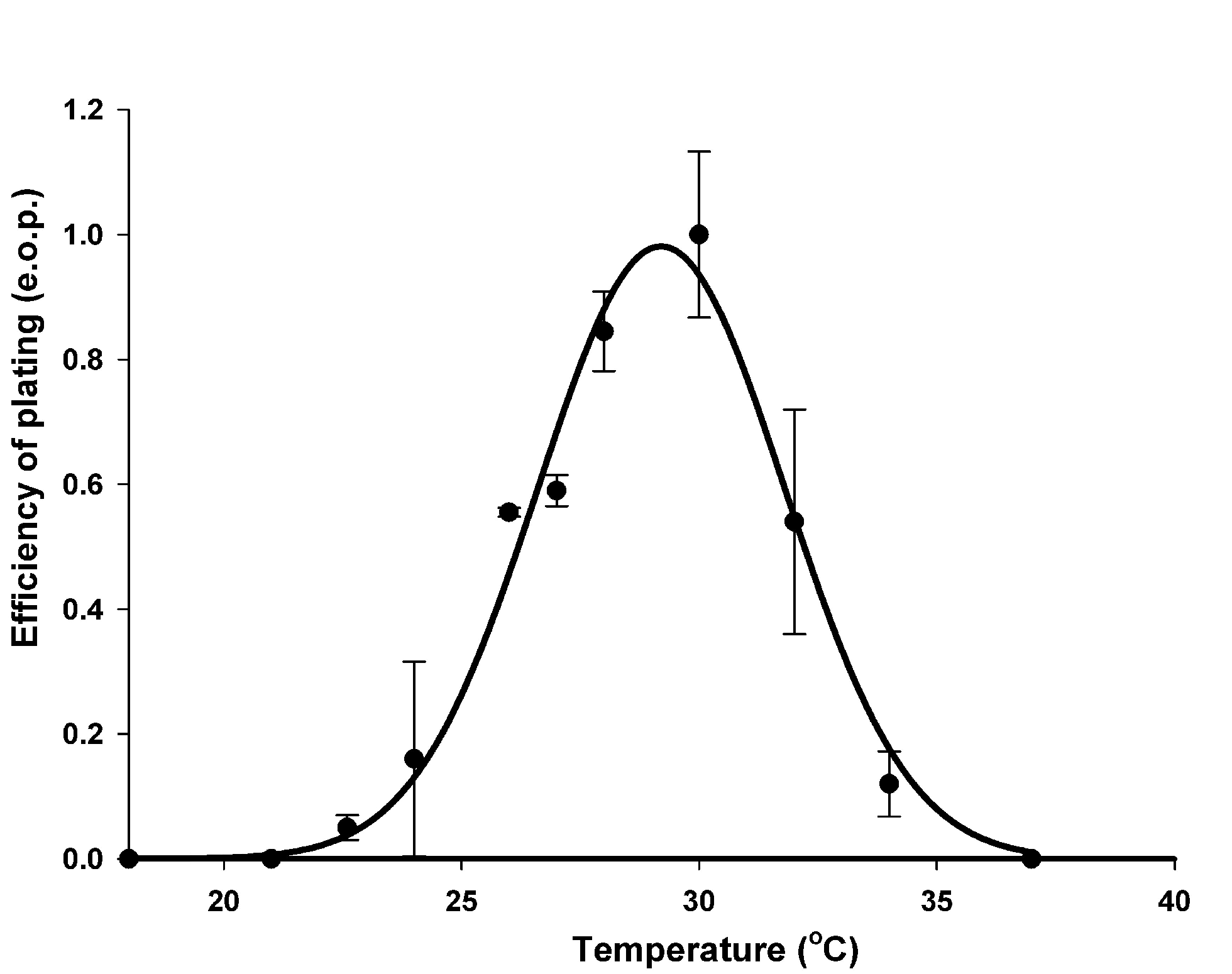

Supplement: Figure S1 — Effect of temperature on the efficiency of plating of phage ArV2. (TIFF) [file pone.0111230.s001.tiff]

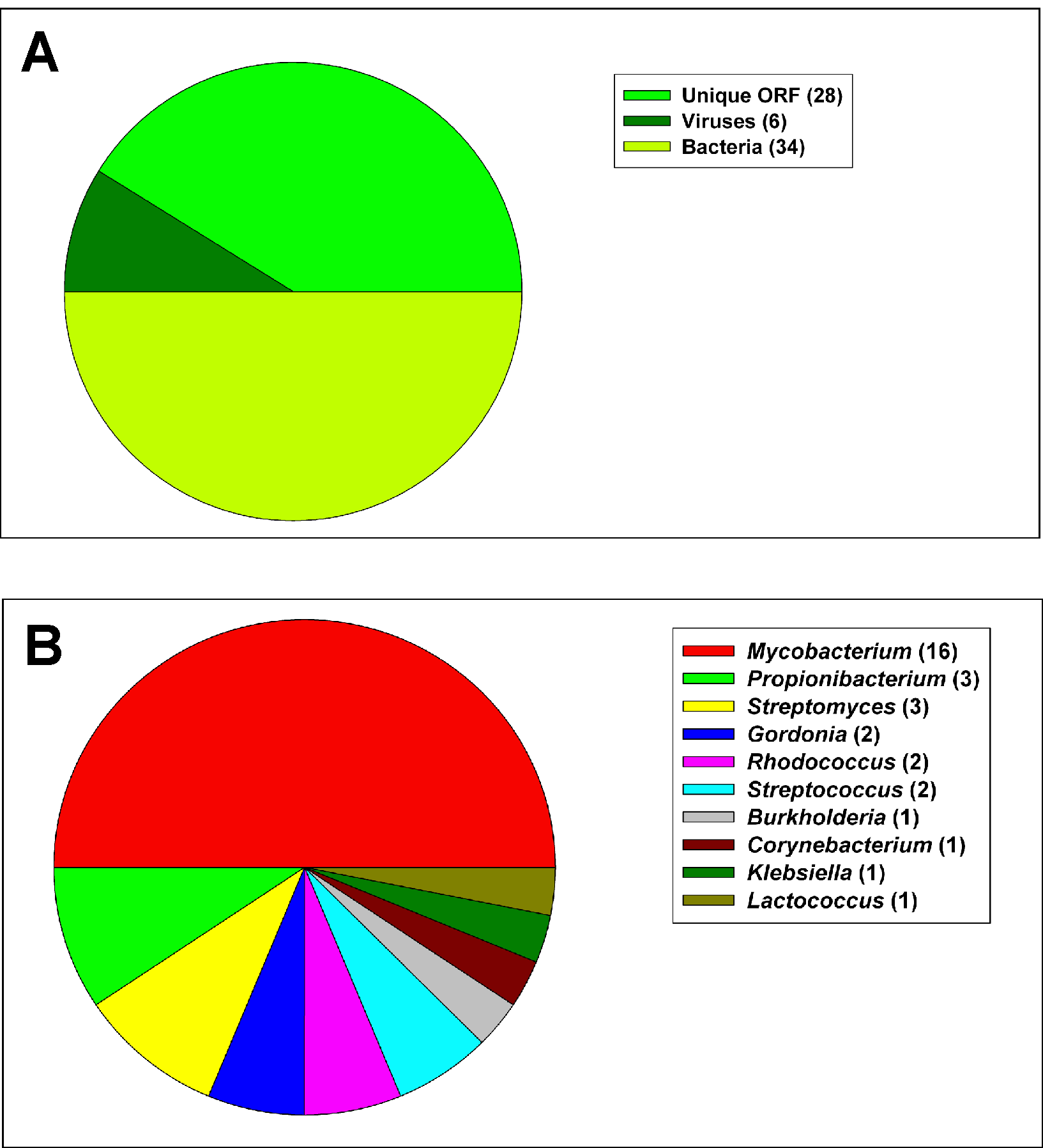

Supplement: Figure S2 — Taxonomy of the best BlastP hits of ArV2 ORFs. (A). The taxonomic distribution of homologues in phages that were grouped according to host specificity (B). (TIFF) [file pone.0111230.s002.tiff]

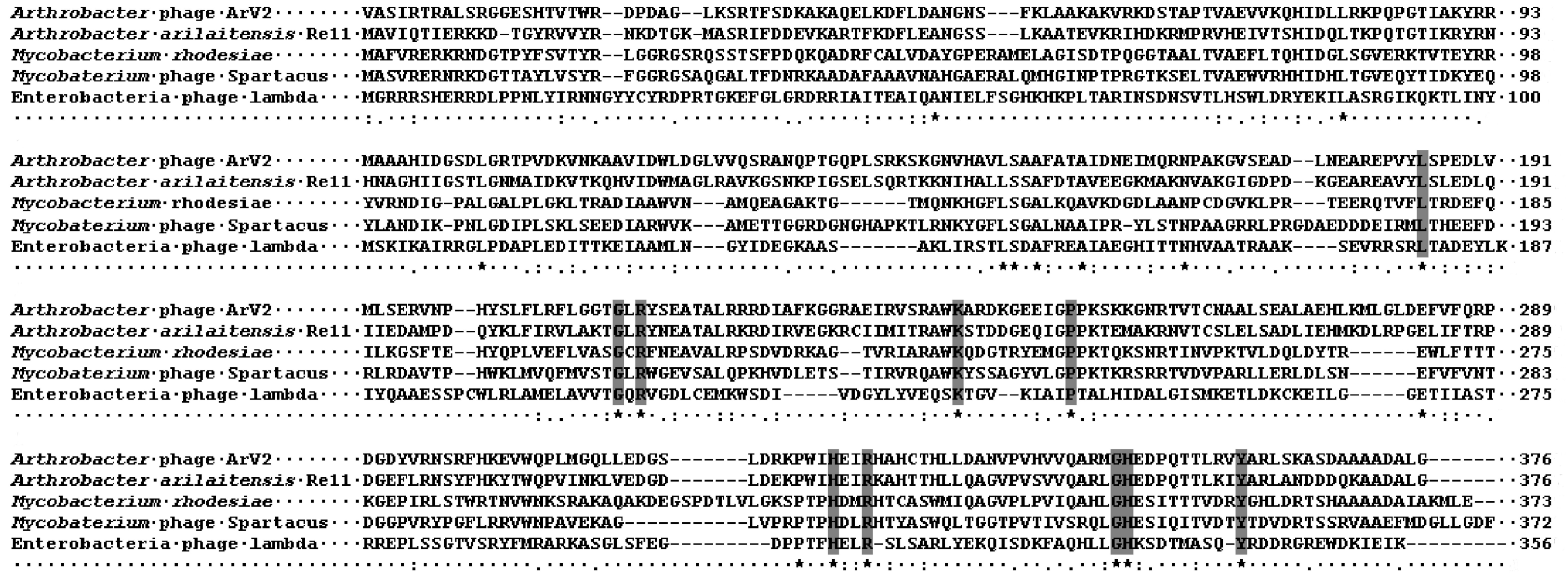

Supplement: Figure S3 — Sequence alignment of the Int family integrase from Arthrobacter phage Arv2 and other organisms. Conserved amino acid positions demonstrated by Nunes-Düby et al. [82] are shaded. (TIFF) [file pone.0111230.s003.tiff]
