# Supplementary material for: Isolation and Characterization of vB_ArS-ArV2 – First Arthrobacter sp. Infecting Bacteriophage with Completely Sequenced Genome
Source: PLoS One. 2014 Oct 21;9(10):e111230. doi: 10.1371/journal.pone.0111230 (PMC4205034; doi:10.1371/journal.pone.0111230)
Supplement: Table S1 — The diversity of Arthrobacter bacteriophages described to date. S – Siphoviridae, P – Podoviridae, nd – not determined. (DOC) [file pone.0111230.s004.doc]

**Table S1. The diversity of *Arthrobacter* bacteriophages described to date.** S – *Siphoviridae*, P – *Podoviridae,* nd – not determined.

| **Name** | **Host** | **Family** | **Morphological characteristics** | **Source and additional information** | **Reference** |
| --- | --- | --- | --- | --- | --- |
|  | *Arthrobacter* sp. | nd | nd | Investigation the use of phages to type soil *Arthrobacter* species. | Conn et al., 1945 |
|  | *Arthrobacter* sp. | nd | nd | Observation of plaques when soil perfusates were plated with *Arthrobacter* sp. | Robinson and Corke, 1959 |
|  | *A. globiformis* strain 616 | nd | nd | Isolation of two phages without studying their morphology. | Gillespie, 1960 |
| *Arthrobacter* bacteriophage | *A. polychromogenes* | S | Hexagonal prismatic head (± 750Å × ± 630Å), the tail sheath with a length of ±2200Å, on which a cross striation is visible (spacing ±35 Å). At the end of the tail sheath an anchor-like structure and no tail fibres were observed. | *Arthrobacter polychromogenes* bacterial culture from airborne infection. | Schippers-Lammertse et al., 1963; Daems, 1963 |
| ϕAG8010 | *A. globiformis* ATCC 8010 | S | Hexagonal head, (69/60 nm), sheatless tail (120/10 nm), suggesting attachment organelle without fibers. | Soil. Slow phage adsorption rate, genomic DNA G+C content 63.3%. | Einck et al., 1973 |
| FX-1 | *A. globiformis* ATCC 8010 | nd | nd | Hagerstown silty clay loam soil. Developement a unique soil enrichment technique for detection of phages in medium, inoculated with soil (with and without additions of host cells). | Casida and Liu, 1974 |
| AC18S-1, AC18S-3, AC18S-4, AC18S-5 | *Arthrobacter* sp. soil isolate AC18 | S | Heads (32–50 nm), tails (85–156 nm). | River water and sewage. | Brown et al., 1978 |
| AC23R-1, AC23S-1, AC23R-3, AC36S-2 | *Arthrobacter* sp. soil isolates AC23, AC36 | S |  | River water. Phage AC23R-3 genomic DNA G+C content 61.2%. Phage AC36S-2 formed pimpoint plaques. |  |
| AC36S-1 | *Arthrobacter* sp. soil isolates AC36, AC23, AN31, AN35, AN39 | S |  | Sewage |  |
| AC36S-3 | *Arthrobacter* sp. soil isolates AC36, AC23, AN31, AN35 | S |  |  |  |
| AN31S-1, AN30S-1 | *Arthrobacter* sp. soil isolates AN31, AN35, AN39, AN25, AN30 | S |  | Sewage. Phage AN31S-1 genomic DNA G+C content 65.3%. |  |
| AC20S-1, AC21S-1, AC30S-1 | *Arthrobacter* sp. soil isolates AC15, AC20, AC21, AC24, AC26, AC29, AC30, 603a, AN21 | S |  | Sewage. Phage AC20S-1 genomic DNA G+C content 60.2%. |  |
| AN29R-2 | *Arthrobacter* sp. soil isolates AN29, AN37 | P | Hexagonal head (40 nm in diameter), short (25 nm) tail, 6 noncontractile tail pins. | Sewage. Phage formed pimpoint plaques. |  |
| AN25S-1 | *Arthrobacter* sp. soil isolates AN31, AN35, AN39, AN25, AN30 | P |  | Sewage. Phage formed 14 mm plaques. |  |
| AN31n-1, AN31n-3 | *Arthrobacter* sp. soil isolate AN 31 | nd | nd | Soil | Ostle and Holt, 1979 |
| AN31n-2 | *Arthrobacter* sp. soil isolate AN 32 | nd | nd |  |  |
| 8010n-1, 8010n-2, 8010c-1 | *Arthrobacter* sp. ATCC 8010 | nd | nd |  |  |
| 15216n-1, 15216c-1 | *Arthrobacter* sp. ATCC 15216 | nd | nd |  |  |
| 11624n-1 | *Arthrobacter* sp. ATCC 11624 | nd | nd |  |  |
| 15841n-1, 15841n-2, 15841n-3 | *Arthrobacter* sp. ATCC 15841 | nd | nd |  |  |
| GAP-15 | *A. globiformis* 8010 | nd | nd | Hagerstown silty clay loam soil. Isolation of *Arthrobacter* bacteriophages from soil using percolation with water and various nutrient solutions. | Germida and Casida, 1981 |
| GAP-16 | *A. globiformis* 8010, *A. globiformis* 4336 | nd | nd |  |  |
| GAP-14, GAP-40 | *A. globiformis* 8010, *A. globiformis* 4336, *Arthrobacter* soil isolate SPI-1 | nd | nd |  |  |
| GAP-41, GAP-42 | *A. oxydans* 14358 | nd | nd |  |  |
| GAP-33 | *Arthrobacter* soil isolate GSI-5 | nd | nd |  |  |
| GAP-32 | *Arthrobacter* soil isolates GSI-5, GSI-1 | nd | nd |  |  |
| GAP-31 | *Arthrobacter* soil isolate GSI-1 | nd | nd |  |  |
| GAP-30 | *Arthrobacter* soil isolates GSI-1, GSI-6 | nd | nd |  |  |
| GAP-34 | *Arthrobacter* soil isolate GSI-6 | nd | nd |  |  |
| AGL1, AGL2, AGL3, AGL4, AGL5, AGL6, AGL8, AGL11, AGL12, AGL13, AGL16, AGL17 | *A. globiformis* CCM 1650 | S | Polyhedral heads (41–57 nm from apex to apex, 43–53 nm from side to side), noncontractile, striated tails (100/7 nm) with a base plate sometimes containing something like a central point. | Soil or vegetation samples from France. Plaques formed by AGL were clear, 2 to 3 mm in diameter, and had a well-defined periphery, except those of AGL4, which had a small (1 mm) clear center surrounded by a turbid edge (2 mm). | Trautwetter and Blanco, 1988 |
| ASP2, ASP7 | *Arthrobacter* sp. strain S155 | S |  | Soil or vegetation samples from France. Plaques slightly turbid, 2-3 mm with a stippled periphery. |  |
| ASP4, ASP16 | *Arthrobacter* sp. strain S155 | S |  | Soil or vegetation samples from France. Plaques slightly turbid, 1-2 mm with a well-defined edge. |  |
| ARA3, ARA8 | *A. ramosus* CCM 1646 | S |  | Soil or vegetation samples from France. Plaques 5 mm with a stippled periphery. |  |
| ARA9 | *A. ramosus* CCM 1646 | S |  | Soil or vegetation samples from France. Plaques 2 mm with a stippled periphery. |  |
| ϕAAU2 | *A. aureus C70* | S | Isometric head (40 nm in diameter), non-contractile tail (150 nm), baseplate containing an 80 nm fibre. | Purification and detailed characterization of temperate phage from samples of soil (all collected from the same industrial site in France). Linear double-stranded DNA of 45.4 kb with cohesive ends according to the restriction analysis of genomic DNA. | Marrec et al., 1994 |
