# Supplementary material for: Isolation and Characterization of vB_ArS-ArV2 – First Arthrobacter sp. Infecting Bacteriophage with Completely Sequenced Genome
Source: PLoS One. 2014 Oct 21;9(10):e111230. doi: 10.1371/journal.pone.0111230 (PMC4205034; doi:10.1371/journal.pone.0111230)
Supplement: Table S2 — ArV2 ORFs with homologues in other viruses or cellular organisms. (DOC) [file pone.0111230.s005.doc]

**Table S2. ArV2 ORFs with homologues in other viruses or cellular organisms.**

| ArV2 ORF,  position | Predicted function  (protein length aa) | Significant match  (protein length aa) | Identity aa %/  similarity aa% (length of the overlapping segment) | | E value |
| --- | --- | --- | --- | --- | --- |
| ORF01  36..380 | hypothetical protein (115) | [WP_010836797.1](http://www.ncbi.nlm.nih.gov/protein/498790051?report=genbank&log$=prottop&blast_rank=2&RID=CNDC4Z0B015) hypothetical protein  *Rhodococcus rhodnii* (101) | | 44/66 (80) | 6e-07 |
|  |  | [NP_569739.1](http://www.ncbi.nlm.nih.gov/protein/18496889?report=genbank&log$=prottop&blast_rank=3&RID=6HT14MA601R) hypothetical protein  *Mycobacterium* phage TM4 (83) | | 44/61  (68) | 0.001 |
| ORF02  349..1803 | terminase (485) | [WP_010836796.1](http://www.ncbi.nlm.nih.gov/protein/498790049?report=genbank&log$=prottop&blast_rank=2&RID=CNDMG3KX015) hypothetical protein  *Rhodococcus rhodnii* (457) | | 48/64 (447) | 9e-138 |
|  |  | [NP_569740.1](http://www.ncbi.nlm.nih.gov/protein/18496890?report=genbank&log$=prottop&blast_rank=2&RID=6HSV9Y2201R) putative terminase gp4  *Mycobacterium* phage TM4 (474) | | 46/63  (456) | 1e-136 |
| ORF03  1797..3194 | portal protein (466) | [WP_010837415.1](http://www.ncbi.nlm.nih.gov/protein/498792554?report=genbank&log$=prottop&blast_rank=2&RID=CNDN7WWX014) hypothetical protein  *Rhodococcus rhodnii* (506) | | 41/57 (435) | 8e-90 |
|  |  | [YP_001936031.1](http://www.ncbi.nlm.nih.gov/protein/189043090?report=genbank&log$=prottop&blast_rank=21&RID=6HT7FJ0E01R) portal protein  *Mycobacterium* phage BPs (504) | | 33/48  (457) | 4e-49 |
| ORF04  3209..4093 | capsid maturation protease (295) | [WP_010837416.1](http://www.ncbi.nlm.nih.gov/protein/498792556?report=genbank&log$=prottop&blast_rank=2&RID=CNDNYM6E015) capsid maturation protease  *Rhodococcus rhodnii* (451) | | 30/46 (253) | 6e-15 |
|  |  | [YP_004678774.1](http://www.ncbi.nlm.nih.gov/protein/338826861?report=genbank&log$=prottop&blast_rank=4&RID=6HT8DR4101R) hypothetical protein  *Gordonia* phage GTE2 (234) | | 34/45  (128) | 3e-09 |
| ORF05  4203..4730 | scaffolding protein (176) | [YP_002241793.1](http://www.ncbi.nlm.nih.gov/protein/206600187?report=genbank&log$=prottop&blast_rank=1&RID=6HTYN7J201R) gp6  *Mycobacterium* phage Ramsey (178) | | 38/58  (126) | 9e-14 |
| ORF06  4761..5657 | major capsid protein (299) | [WP_017619396.1](http://www.ncbi.nlm.nih.gov/protein/516215433?report=genbank&log$=prottop&blast_rank=2&RID=CNDWARXM014) hypothetical protein  *Nocardiopsis gilva* (311) | | 61/74 (304) | 6e-111 |
|  |  | [YP_008051783.1](http://www.ncbi.nlm.nih.gov/protein/508179836?report=genbank&log$=prottop&blast_rank=4&RID=6HTZEX7001R) major capsid protein  *Mycobacterium* phage Severus (310) | | 50/65  (310) | 7e-91 |
| ORF07  5708..6289 | hypothetical protein (194) | [WP_004015827.1](http://www.ncbi.nlm.nih.gov/protein/490115224?report=genbank&log$=prottop&blast_rank=2&RID=CNDWW7PK015) hypothetical protein  *Mobiluncus mulieris* (135) | | 47/63 (107) | 8e-25 |
|  |  | [NP_046834.1](http://www.ncbi.nlm.nih.gov/protein/9630402?report=genbank&log$=prottop&blast_rank=2&RID=6HU01P3S01R) gp19  *Mycobacterium* phage D29 (124) | | 30/52  (112) | 3e-06 |
| ORF08  6286..6657 | hypothetical protein (124) | [WP_010696612.1](http://www.ncbi.nlm.nih.gov/protein/498382456?report=genbank&log$=prottop&blast_rank=1&RID=6HUJKF4201R) hypothetical protein  *Saccharopolyspora spinosa* (110) | | 32/53  (84) | 7e-07 |
| ORF10  6953..7360 | hypothetical protein (136) | [WP_004015830.1](http://www.ncbi.nlm.nih.gov/protein/490115227?report=genbank&log$=prottop&blast_rank=1&RID=6HUK9WUX01R) hypothetical protein  *Mobiluncus mulieris* (135) | | 29/49  (138) | 2e-07 |
| ORF11  7388..8074 | major tail protein (229) | [WP_006548213.1](http://www.ncbi.nlm.nih.gov/protein/493595457?report=genbank&log$=prottop&blast_rank=3&RID=CNDXMGPB015) major tail protein  *Actinomyces urogenitalis* (221) | | 44/63 (221) | 3e-57 |
|  |  | [YP_001468940.1](http://www.ncbi.nlm.nih.gov/protein/157310944?report=genbank&log$=prottop&blast_rank=7&RID=6HUM2SMD01R) putative major tail protein *Corynebacterium* phage P1201 (225) | | 29/46  (218) | 6e-20 |
| ORF13  8348..8689 | hypothetical protein (114) | [WP_021604833.1](http://www.ncbi.nlm.nih.gov/protein/545331190?report=genbank&log$=prottop&blast_rank=1&RID=6HUUH93U01R) hypothetical protein  *Actinomyces johnsonii* (111) | | 42/59  (111) | 7e-14 |
| ORF14  8743..9132 | hypothetical protein (130) | [WP_004015833.1](http://www.ncbi.nlm.nih.gov/protein/490115230?report=genbank&log$=prottop&blast_rank=2&RID=CNE30Z9P014) hypothetical protein  *Mobiluncus mulieris* (136) | | 38/52 (124) | 3e-15 |
|  |  | [YP_004935759.1](http://www.ncbi.nlm.nih.gov/protein/359802293?report=genbank&log$=prottop&blast_rank=4&RID=6HUV44PB01R) unnamed protein product  *Gordonia* phage GTE5 (270) | | 25/40  (142) | 0.035 |
| ORF15  9152..12778 | tape measure protein (1209) | [WP_010549680.1](http://www.ncbi.nlm.nih.gov/protein/498235524?report=genbank&log$=prottop&blast_rank=2&RID=CNE3JDU0015) hypothetical protein  *Brachybacterium paraconglomeratum* (1495) | | 34/48 (1146) | 2e-140 |
|  |  | [ADD81132.1](http://www.ncbi.nlm.nih.gov/protein/291165105?report=genbank&log$=prottop&blast_rank=2&RID=6HUW29EK01R) tape measure protein  *Rhodococcus* phage ReqiPine5 (1732) | | 35/49  (950) | 3e-122 |
| ORF16  12791..13762 | tail protein (324) | [WP_018188152.1](http://www.ncbi.nlm.nih.gov/protein/516966025?report=genbank&log$=prottop&blast_rank=2&RID=CNE48E9A015) hypothetical protein  *Microbacterium paraoxydans* (301) | | 33/48 (327) | 1e-37 |
|  |  | [YP_006906508.1](http://www.ncbi.nlm.nih.gov/protein/410491286?report=genbank&log$=prottop&blast_rank=3&RID=6HVDSEU901R) putative minor tail protein  *Propionibacterium* phage P100_1 (313) | | 30/46  (296) | 4e-26 |
| ORF17  13763..14869 | tail protein (369) | [YP_006906556.1](http://www.ncbi.nlm.nih.gov/protein/410491334?report=genbank&log$=prottop&blast_rank=1&RID=6HVEPKKJ01R) putative minor tail protein *Propionibacterium* phage P101A (385) | | 33/48  (349) | 6e-42 |
| ORF18  14869..15855 | tail protein (329) | [WP_018188150.1](http://www.ncbi.nlm.nih.gov/protein/516966021?report=genbank&log$=prottop&blast_rank=2&RID=CNEDW356014) hypothetical protein  *Microbacterium paraoxydans* (640) | | 36/55 (165) | 6e-22 |
|  |  | [YP_006907146.1](http://www.ncbi.nlm.nih.gov/protein/410491878?report=genbank&log$=prottop&blast_rank=5&RID=6HVFMTDY01R) putative minor tail protein *Propionibacterium* phage P101A (272) | | 32/47  (124) | 0.008 |
| ORF20  16169..16945 | tail fiber protein (259) | [YP_719837.1](http://www.ncbi.nlm.nih.gov/protein/113461768?report=genbank&log$=prottop&blast_rank=3&RID=CNERTMHS014) large adhesin  *Haemophilus somnus* 129PT (1386) | | 58/62 (111) | 3e-18 |
|  |  | [AEL17839.1](http://www.ncbi.nlm.nih.gov/protein/342221245?report=genbank&log$=prottop&blast_rank=40&RID=6HWK623R01R) gp7  *Mycobacterium* phage Trixie (316) | | 47/58  (129) | 3e-14 |
| ORF21  16864..17796 | peptidase (311) | [WP_021470902.1](http://www.ncbi.nlm.nih.gov/protein/545107816?report=genbank&log$=prottop&blast_rank=1&RID=6HWKT3Y701R) hypothetical protein  *Arthrobacter* sp. AK-YN10 (272) | | 51/63  (230) | 3e-66 |
|  |  | [ACU41885.1](http://www.ncbi.nlm.nih.gov/protein/255928268?report=genbank&log$=prottop&blast_rank=2&RID=CN9UN2XZ014) gp48  *Mycobacterium* phage Puhltonio (438) | | 38/46  (154) | 1e-14 |
| ORF24  18754..20241 | tail fiber protein (496) | [YP_002782645.1](http://www.ncbi.nlm.nih.gov/protein/226364863?report=genbank&log$=prottop&blast_rank=1&RID=6HWMNC3T01R) hypothetical protein *Rhodococcus* *opacus* B4 (275) | | 32/46  (211) | 4e-17 |
| ORF26  20690..21238 | HNH endonuclease (183) | [YP_003347628.1](http://www.ncbi.nlm.nih.gov/protein/282554611?report=genbank&log$=prottop&blast_rank=2&RID=6HXAKBHE01R) hypothetical protein  *Klebsiella* phage KP34 (136) | | 38/50  (117) | 9e-14 |
| ORF27c  (21378..21235) | hypothetical protein (48) | [WP_005052671.1](http://www.ncbi.nlm.nih.gov/protein/491194321?report=genbank&log$=prottop&blast_rank=1&RID=6HXBE40Y01R) hypothetical protein  *Microbacterium laevaniformans* (50) | | 56/73  (45) | 2e-07 |
| ORF28  21433..21702 | hypothetical protein (90) | [YP_002488005.1](http://www.ncbi.nlm.nih.gov/protein/220912696?report=genbank&log$=prottop&blast_rank=1&RID=6HXC9FXV01R) hypothetical protein  *Arthrobacter chlorophenolicus* A6 (90) | | 49/57  (68) | 6e-07 |
| ORF29c  (22829..21699) | phage integrase (377) | [YP_003916402.1](http://www.ncbi.nlm.nih.gov/protein/308176996?report=genbank&log$=prottop&blast_rank=1&RID=6HXMRS5M01R) phage integrase  *Arthrobacter arilaitensis* Re117 (376) | | 54/69  (362) | 5e-135 |
|  |  | [AFA45049.1](http://www.ncbi.nlm.nih.gov/protein/375281875?report=genbank&log$=prottop&blast_rank=2&RID=CNA9933S015) gp41  *Mycobacterium* phage Spartacus (372) | | 32/48  (310) | 2e-36 |
| ORF31  23128..23586 | hypothetical protein (153) | [YP_655323.1](http://www.ncbi.nlm.nih.gov/protein/109521886?report=genbank&log$=prottop&blast_rank=1&RID=6HXTKWVS01R) gp46  *Mycobacterium* phage Pipefish (161) | | 33/45  (118) | 0.12 |
| ORF34c  (24381..23989) | phage repressor protein (131) | [WP_016663149.1](http://www.ncbi.nlm.nih.gov/protein/514974944?report=genbank&log$=prottop&blast_rank=2&RID=CNEF6CZ6015) hypothetical protein  *Dermabacter* sp. HFH0086 (146) | | 34/51 (94) | 8e-05 |
|  |  | [NP_049993.1](http://www.ncbi.nlm.nih.gov/protein/9632965?report=genbank&log$=prottop&blast_rank=9&RID=6HXPFTAF01R) cI-like repressor  *Streptococcus* phage Sfi21 (127) | | 42/58  (55) | 0.002 |
| ORF35  24447..24674 | putative transcriptional regulator (76) | [GAD35287.1](http://www.ncbi.nlm.nih.gov/protein/537393442?report=genbank&log$=prottop&blast_rank=1&RID=6HY6KGXB01R) putative transcriptional regulator *Microbacterium* sp. TS-1 (89) | | 36/62  (66) | 2e-04 |
| ORF38  25214..25639 | transcriptional factor WhiB-like (142) | [YP_579189.1](http://www.ncbi.nlm.nih.gov/protein/93007425?report=genbank&log$=prottop&blast_rank=1&RID=6HY7CHWB01R) transcriptional factor WhiB-like *Streptomyces* phage mu1/6 (170) | | 37/51  (98) | 1e-07 |
| ORF39  25620..25892 | hypothetical protein (91) | [WP_007271583.1](http://www.ncbi.nlm.nih.gov/protein/494482108?report=genbank&log$=prottop&blast_rank=1&RID=6HY832JM01R) hypothetical protein  *Arthrobacter gangotriensis* (93) | | 47/55  (76) | 2e-11 |
| ORF52  29030..29857 | Exonuclease (276) | [YP_006668139.1](http://www.ncbi.nlm.nih.gov/protein/404213945?report=genbank&log$=prottop&blast_rank=2&RID=CNEZ906M014) hypothetical protein KTR9_1344  *Gordonia* sp. KTR9 (463) | | 43/59 (253) | 6e-53 |
|  |  | [YP_655559.1](http://www.ncbi.nlm.nih.gov/protein/109392329?report=genbank&log$=prottop&blast_rank=19&RID=6HZ1CZD201R) gp42  *Mycobacterium* phage Halo (361) | | 32/50  (286) | 4e-35 |
| ORF53  29854..30420 | hypothetical protein (189) | [WP_020473353.1](http://www.ncbi.nlm.nih.gov/protein/521961748?report=genbank&log$=prottop&blast_rank=2&RID=CNF01PSA015) hypothetical protein  *Zavarzinella formosa* (193) | | 43/60 (164) | 9e-32 |
|  |  | [YP_005087102.1](http://www.ncbi.nlm.nih.gov/protein/372449879?report=genbank&log$=prottop&blast_rank=3&RID=6HZ21C3301R) unnamed protein product  *Rhodococcus* phage REQ2 (207) | | 50/66  (124) | 7e-31 |
| ORF54  30423..30827 | ssDNA binding protein (135) | [YP_003162086.1](http://www.ncbi.nlm.nih.gov/protein/256833359?report=genbank&log$=prottop&blast_rank=2&RID=CNF0PZM7014) single-strand binding protein  *Jonesia denitrificans* DSM 20603 (137) | | 42/52 (142) | 2e-21 |
|  |  | [YP_008408740.1](http://www.ncbi.nlm.nih.gov/protein/530545136?report=genbank&log$=prottop&blast_rank=45&RID=6HZ4YKA501R) ssDNA binding protein  *Mycobacterium* phage Whirlwind(127) | | 35/50  (127) | 9e-09 |
| ORF57  31418..31795 | transcriptional factor WhiB-like (126) | [WP_021470856.1](http://www.ncbi.nlm.nih.gov/protein/545107770?report=genbank&log$=prottop&blast_rank=2&RID=CNF50WTH014) hypothetical protein M707_02545  *Arthrobacter* sp. AK-YN10 (74) | | 53/63 (38) | 4e-05 |
|  |  | [YP_579189.1](http://www.ncbi.nlm.nih.gov/protein/93007425?report=genbank&log$=prottop&blast_rank=8&RID=6HZFUR3601R) transcriptional factor WhiB-like *Streptomyces* phage mu1/6 (170) | | 46/58  (39) | 0.25 |
| ORF58  31792..32118 | hypothetical protein (109) | [AFL47985.1](http://www.ncbi.nlm.nih.gov/protein/390098557?report=genbank&log$=prottop&blast_rank=1&RID=6HZGDCAU01R) gp80  *Mycobacterium* phage Avani (184) | | 35/52  (74) | 4e-05 |
| ORF59  32108..32857 | hypothetical replication protein (250) | [WP_021470860.1](http://www.ncbi.nlm.nih.gov/protein/545107774?report=genbank&log$=prottop&blast_rank=3&RID=CNF5PS27014) hypothetical protein M707_02565  *Arthrobacter* sp. AK-YN10 (258) | | 29/46 (242) | 3e-14 |
|  |  | [NP_958257.1](http://www.ncbi.nlm.nih.gov/protein/41057231?report=genbank&log$=prottop&blast_rank=26&RID=6HZH1TNS01R) hypothetical protein  *Streptomyces* phage VWB (318) | | 32/47  (123) | 3e-09 |
| ORF60  32833..33102 | hypothetical protein (90) | [YP_004906048.1](http://www.ncbi.nlm.nih.gov/protein/357391207?report=genbank&log$=prottop&blast_rank=2&RID=CNF6B8MW015) hypothetical protein KSE_43085  *Kitasatospora setae* KM-6054 (74) | | 44/51 (62) | 4e-06 |
|  |  | [AER47990.1](http://www.ncbi.nlm.nih.gov/protein/354996646?report=genbank&log$=prottop&blast_rank=2&RID=6HZW1PEA01R) gp139  *Mycobacterium* phage Courthouse (90) | | 52/64  (42) | 0.003 |
| ORF61  33084..34412 | DNA helicase (443) | [WP_007265258.1](http://www.ncbi.nlm.nih.gov/protein/494475781?report=genbank&log$=prottop&blast_rank=1&RID=6J2XFTJ1014) DNA helicase  *Streptomyces* sp. C (493) | | 42/62  (434) | 4e-104 |
|  |  | [YP_008408726.1](http://www.ncbi.nlm.nih.gov/protein/530545122?report=genbank&log$=prottop&blast_rank=2&RID=CNAA7PF6015) DNA helicase  Mycobacterium phage Whirlwind (421) | | 37/54  (425) | 1e-70 |
| ORF64  34794..35303 | DNA N-6-adenine-methyltransferase (170) | [WP_016464768.1](http://www.ncbi.nlm.nih.gov/protein/512641493?report=genbank&log$=prottop&blast_rank=2&RID=CNFAZXWK015) hypothetical protein  *Microbacterium* sp. oral taxon 186 (176) | | 66/72 (163) | 5e-62 |
|  |  | [YP_355379.1](http://www.ncbi.nlm.nih.gov/protein/77864669?report=genbank&log$=prottop&blast_rank=3&RID=6HZXMGY901R) gp44  *Burkholderia* phage Bcep176 (157) | | 58/67  (151) | 8e-53 |
| ORF65  35300..35896 | putative endodeoxyribonuclease (199) | [WP_004016745.1](http://www.ncbi.nlm.nih.gov/protein/490116156?report=genbank&log$=prottop&blast_rank=2&RID=CNFBWC1V015) hypothetical protein  *Mobiluncus mulieris* (131) | | 35/50 (106) | 7e-10 |
|  |  | [NP_817741.1](http://www.ncbi.nlm.nih.gov/protein/29566170?report=genbank&log$=prottop&blast_rank=14&RID=6J02GC5R01R) gp64  *Mycobacterium* phage Che9c (127) | | 34/48  (93) | 0.005 |
| ORF67c  (36996..36658) | hypothetical protein (113) | [WP_019299137.1](http://www.ncbi.nlm.nih.gov/protein/518128929?report=genbank&log$=prottop&blast_rank=2&RID=CNDAJEB0015) hypothetical protein  *Lactococcus garvieae* (232) | | 50/60 (94) | 1e-10 |
|  |  | [NP_076697.1](http://www.ncbi.nlm.nih.gov/protein/13095807?report=genbank&log$=prottop&blast_rank=4&RID=6HSKHHZZ01R) Orf2  *Lactococcus* phage bIL309 (192) | | 44/59 (105) | 5e-10 |
| ORF68  37011..37319 | HNH endonuclease (103) | [WP_007058744.1](http://www.ncbi.nlm.nih.gov/protein/494118965?report=genbank&log$=prottop&blast_rank=3&RID=CNDB8SPW015) HNH endonuclease  *Bfidobacterium longum* subsp*. longum* CECT 7347 (117) | | 50/57 (111) | 9e-23 |
|  |  | [AGF87443.1](http://www.ncbi.nlm.nih.gov/protein/451937137?report=genbank&log$=prottop&blast_rank=23&RID=6HSU0D1201R) putative HNH endonuclease  *Streptococcus* phage phi5218 (120) | | 40/61 (70) | 2e-10 |
